# Cpeb1b-mediated cytoplasmic polyadenylation of *shha* mRNA modulates zebrafish definitive hematopoiesis

**DOI:** 10.1073/pnas.2212212120

**Published:** 2023-02-06

**Authors:** Jian Heng, Boyang Shi, Jia-Yi Zhou, Yifan Zhang, Dongyuan Ma, Yun-Gui Yang, Feng Liu

**Affiliations:** ^a^State Key Laboratory of Membrane Biology, Institute of Zoology, Chinese Academy of Sciences, Beijing 100101, China; ^b^Institute for Stem Cell and Regeneration, Chinese Academy of Sciences, Beijing 100101, China; ^c^CAS Key Laboratory of Genomic and Precision Medicine, Collaborative Innovation Center of Genetics and Development, College of Future Technology, Beijing Institute of Genomics, Chinese Academy of Sciences and China National Center for Bioinformation, Beijing 100101, China; ^d^University of Chinese Academy of Sciences, Beijing 101408, China; ^e^School of Life Sciences, Shandong University, Qingdao 266237, China

**Keywords:** hematopoietic stem and progenitor cell, cytoplasmic polyadenylation, Cpeb1b, Hedgehog signaling, translational control

## Abstract

During vertebrate embryogenesis, hematopoietic stem and progenitor cell (HSPC) production through endothelial-to-hematopoietic transition requires suitable developmental signals, but how these signals are accurately regulated remains incompletely understood. Cytoplasmic polyadenylation, which is one of the posttranscriptional regulations, plays a crucial role in RNA metabolism. Here, we report that Cpeb1b-mediated cytoplasmic polyadenylation is important for HSPC specification by translational control of Hedgehog (Hh) signaling during zebrafish early development. Cpeb1b is highly expressed in notochord and its deficiency results in defective HSPC production. Mechanistically, Cpeb1b regulates hemogenic endothelium specification by the Hedgehog–Vegf–Notch axis. We demonstrate that the cytoplasmic polyadenylation element motif-dependent interaction between Cpeb1b and *shha* messenger RNA (mRNA) in the liquid-like condensates, which are induced by Pabpc1b phase separation, is required for cytoplasmic polyadenylation of *shha* mRNA. Intriguingly, the cytoplasmic polyadenylation regulates translation but not stability of *shha* mRNA, which further enhances the Shha protein level and Hh signal transduction. Taken together, our findings uncover the role of Cpeb1b-mediated cytoplasmic polyadenylation in HSPC development and provide insights into how posttranscriptional regulation can direct developmental signals with high fidelity to translate them into cell fate transition.

## Significance

HSPCs have the capacities of both self-renewal and multilineage differentiation and hold great importance for lifelong blood supply. The developmental signals ensure the production of HSPCs through endothelial-to-hematopoietic transition during vertebrate embryogenesis. However, how these signals are accurately regulated to execute cell fate transition remains elusive. In this study, we explore the role of Cpeb1b in HSPC production and uncover its function via cytoplasmic polyadenylation of *shha* mRNA in this process. We show that notochord-expressed Cpeb1b is required for HSPC development by regulating Hedgehog signaling. Cpeb1b interacts with *shha* mRNA in the liquid-like condensates and promotes its cytoplasmic polyadenylation. Cytoplasmic polyadenylation of *shha* mRNA enhances its own translation efficiency and therefore increases the Shha protein level, which facilitates Hedgehog signaling activation.

During vertebrate embryonic development, the hematopoiesis occurs through two waves: the primitive wave only generates erythrocytes and myelocytes, and the definitive wave can produce hematopoietic stem and progenitor cells (HSPCs), which have the capacity to give rise to all lineages of blood cells and maintain the entire blood system (1–3). During the definitive hematopoiesis period, a subset of endothelial cells (ECs) receive cell-intrinsic and cell-extrinsic signals and acquire hemogenic potential to become definitive hemogenic endothelium (HE) cells in the mammalian aorta-gonad-mesonephros region or the zebrafish ventral wall of the dorsal aorta (VDA) region. Then, these HE cells gradually change their shape and bud from the dorsal aorta (DA) to be converted into HSPCs through the endothelial-to-hematopoietic transition (4–7). However, how these ECs are precisely controlled to undergo EC-to-HSPC switch remains incompletely understood.

Several developmental signals have been reported to be critical for hematopoiesis (2, 3, 8). Among these signals, Hedgehog (Hh) signaling is shown to be involved in HSPC production in zebrafish and mammals (9–11). In the Hh signaling transduction process, the secreted signal moiety, which is generated by auto-cleavage of Hh precursor, binds to Patched receptor, leading to disinhibition and primary cilium tip location of Smoothened (Smo). Then, the activated Smo facilitates the dissociation of Gli2/3-Cos-SuFu complex, resulting in release of the full-length forms of Gli2/3, which function as activators for Hh downstream gene transcription. Hh downstream genes can indirectly induce Notch signaling through upregulation of Vegf signaling (1, 10, 11). Upon Notch activation, Notch intracellular domain (NICD) enters the nucleus to activate transcription of Notch target genes, which are required for HE specification (12–14). However, how these signals are accurately regulated during HSPC production should be further explored.

The posttranscriptional regulation, including alternative splicing, noncoding RNA-mediated regulation, RNA modification, poly(A) tail length, codon usage, and RNA structure, plays a crucial role in the regulation of gene expression and signaling specificity through controlling metabolism of RNA molecules (15–20). Recently, the role of posttranscriptional regulation in hematopoiesis continues to be reported. The posttranscriptional regulators, such as ADAR1 (21), miR-142a-3p (18), Rbm15 (22), Regnase-1 (23), and m^6^A modification (16), are involved in HSPC production or maintenance through different influences on RNA metabolism. Cytoplasmic polyadenylation as one of the posttranscriptional regulations is mainly regulated by the cytoplasmic polyadenylation element binding protein (CPEB) family proteins (24, 25). CPEB specifically binds to the U-rich cytoplasmic polyadenylation element (CPE) in 3′ untranslated region (UTR) of target mRNAs and nucleates a cytoplasmic complex containing the noncanonical poly(A) polymerase Tent2/Papd4/Gld2. Tent2/Papd4/Gld2 can add successive adenosine 5'-monophosphate monomers to the 3′-end of mRNAs for poly(A) tail formation, further facilitating mRNA circularization and translation initiation (20, 25–27). CPEB proteins are involved in regulation of diverse biological processes, including synaptic plasticity and long-term potentiation (28–31), germ cell differentiation (32), cellular senescence (33), muscle stem cell activation (34), and immune response (35). However, it remains unknown whether CPEB proteins play a role in regulating developmental signals that affect embryonic HSPC production in vertebrates.

Due to genome duplication in teleost fish, there are two CPEB1 orthologs in zebrafish, *cpeb1a* and *cpeb1b* (36). Here, we have demonstrated that Cpeb1b-mediated cytoplasmic polyadenylation is important for HSPC development in zebrafish. Cpeb1b deficiency leads to HE specification defect. Further mechanistic studies show that Cpeb1b interacts with *shha* mRNA in the liquid-like condensates, which are induced by Pabpc1b phase separation. This interaction is CPE motif-dependent and crucial for cytoplasmic polyadenylation of *shha* mRNA. Cpeb1b-regulated cytoplasmic polyadenylation of *shha* mRNA promotes its translation and further facilitates Hedgehog–Vegf–Notch signaling axis activation, which are required for HSPC production.

## Results

### Cpeb1b is Important for HSPC Production during Early Development.

**Fig. 1.**
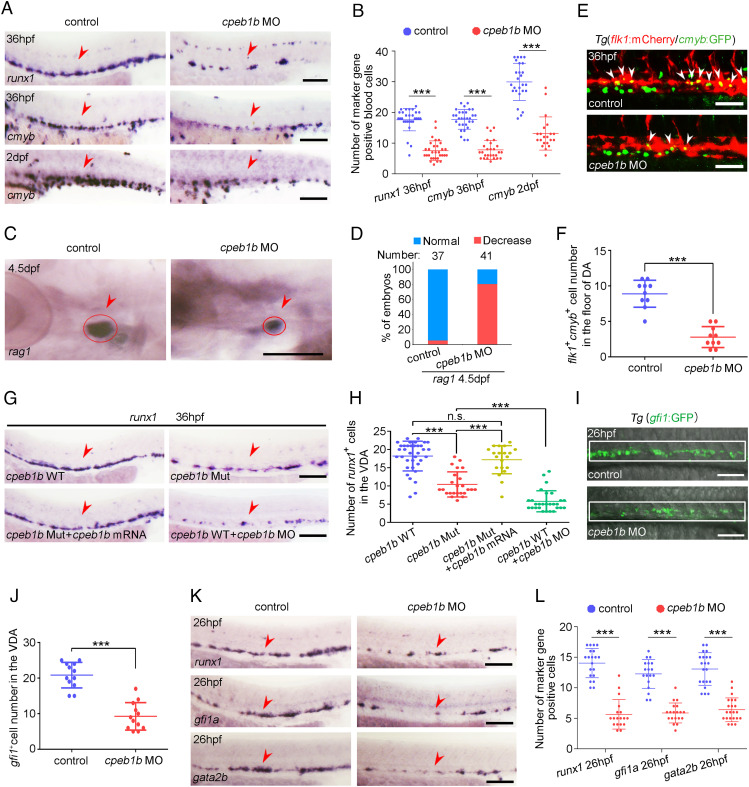
Cpeb1b deficiency impairs HSPC production. (*A*) Examination of the expression of *runx1* and *cmyb* in the control and *cpeb1b* morphants by WISH. The red arrowheads denote HSPCs. (Scale bar, 100 μm.) (*B*) Statistical analysis of the WISH. Error bar, mean ± SD, ***P < 0.001. (*C*) Examination of the *rag1* expression in the control and *cpeb1b* morphants by WISH. (*D*) Quantification of the WISH. (*E*) Confocal imaging shows the *flk1*^+^*cmyb*^+^ definitive hematopoietic precursors in the VDA region of control and *cpeb1b* morphants at 36 hpf. White arrowheads denote *flk1*^+^*cmyb*^+^ precursors. (Scale bars, 100 μm.) (*F*) Statistical analysis of *flk1*^+^*cmyb*^+^ definitive hematopoietic precursors. Error bar, mean ± SD. The *P* value was calculated by Student’s *t* test, ***P < 0.001. (*G*) Examination of the *runx1* expression in WT, *cpeb1b* mutant, *cpeb1b* mRNA-overexpressed *cpeb1b* mutant, and *cpeb1b* MO-injected WT embryos by WISH. In WISH experiments using *cpeb1b* mutant embryos, the embryos were obtained by cross-mating *cpeb1b* adult mutants and individually subjected to genotyping after WISH for identifying homozygous mutants. (Scale bar, 100 μm.) (*H*) Statistical analysis of the WISH. Error bar, mean ± SD, n.s.: no significance, ***P < 0.001. (*I*) Confocal imaging shows the *gfi1*^+^ HE cells in the VDA region of control and *cpeb1b* morphants at 26 hpf. (Scale bars, 100 μm.) (*J*) Statistical analysis of *gfi1*^+^ HE cells. Error bar, mean ± SD. The *P* value was calculated by Student’s *t* test, ***P < 0.001. (*K*) Examination of the HE marker *runx1*, *gfi1a*, and *gata2b* expression in control and *cpeb1b* morphants at 26 hpf by WISH. (Scale bars, 100 μm.) (*L*) Statistical analysis of the WISH. Error bar, mean ± SD, ***P < 0.001.

To examine the expression pattern of *cpeb1b* during zebrafish early development, we performed whole-mount in situ hybridization (WISH) and found that *cpeb1b* displayed maternal expression and was highly expressed in the notochord region from 14 to 24 h post fertilization (hpf) (*SI Appendix*, Fig. S1*A*). Moreover, double fluorescence in situ hybridization (FISH) was carried out with *cpeb1b* and a well-known notochord-specific marker gene *shha*. FISH result showed that the green *cpeb1b* signals were colocalized with the red *shha* signals in the notochord at 14 and 16 hpf (*SI Appendix*, Fig. S1*B*), confirming the notochord*-*enriched expression of *cpeb1b*. To determine whether Cpeb1b plays a potential role in zebrafish embryonic hematopoiesis, we utilized the knockdown approach by injection of *cpeb1b* anti-sense ATG morpholino (MO) (*SI Appendix*, Fig. S2*A*), whose effectiveness was verified by western blot (WB) (*SI Appendix*, Fig. S2 *B* and *C*). WISH revealed that the primitive hematopoiesis marked by *gata1* (erythroid) and *spi1* (myeloid) expression at 14 hpf was normal (*SI Appendix*, Fig. S2 *D* and *E*), and time-lapse imaging showed that the blood flow at 32 hpf was not altered in the *cpeb1b* morphants (MO-injected embryos) compared with control (Movie S1). However, *cpeb1b* morphants exhibited impaired HSPC production in definitive hematopoiesis marked by the expression of HSPC markers *runx1* and *cmyb* in VDA at 36 hpf (Fig. 1 *A* and *B*). The number of *cmyb*-marked HSPCs in CHT at 2 days post fertilization (dpf) and HSPC-derived lymphocytes in thymus at 4.5 dpf was also evidently reduced in the *cpeb1b* morphants (Fig. 1 *A*–*D*). Notably, overexpression of *cpeb1b* mRNA without the *cpeb1b* MO binding site (to avoid inhibition by its antisense MO) (*SI Appendix*, Fig. S2 *F* and *G*) rescued this HSPC defect (*SI Appendix*, Fig. S2 *H* and *I*), indicating that the defective phenotype resulted from *cpeb1b* loss-of-function. To further validate the HSPC phenotype upon *cpeb1b* knockdown, we observed the living definitive hematopoietic precursors by using the Tg(*flk1*:mCherry/*cmyb*:GFP) double-transgenic line and found that the number of *flk1* and *cmyb* double-positive precursors within the VDA region was markedly decreased in the *cpeb1b* morphants (Fig. 1 *E* and *F*). We performed further flow cytometry analysis and found similar results (*SI Appendix*, Fig. S2 *J* and *K*). In addition, we generated a *cpeb1b*-null frameshift mutant by CRISPR/Cas9 technology to confirm the *cpeb1b* loss-of-function phenotype. The zygotic mutant embryos were obtained by cross-mating heterozygous mutants (*SI Appendix*, Fig. S2*L*), however, we failed to obtain the maternal-zygotic mutant embryos due to the defective germ cell and ovary development in zygotic mutant adult zebrafish, which are similar to that in mouse (32). WISH showed that the HSPC production was impaired in *cpeb1b* zygotic mutant, and this phenotype was also rescued by *cpeb1b* mRNA overexpression (Fig. 1 *G* and *H*), supporting that the phenotype is not due to the off-target effect of CRISPR/Cas9. However, the HSPC phenotype in *cpeb1b* zygotic mutant was milder than that in the *cpeb1b* morphants (Fig. 1 *G* and *H*). Previous studies showed that the maternal effect in the zygotic mutant may alleviate the phenotype, and thus, suppressing this maternal effect by a low dose of MO injection leads to more severe phenotype (37, 38). Based on the finding of *cpeb1b* maternal expression, we wondered whether the maternal effect may lead to alleviative phenotype in *cpeb1b* zygotic mutant. To test this hypothesis, we injected low-dose (LD) *cpeb1b* MO into wild-type (WT) and *cpeb1b* zygotic mutants and observed severe HSPC defect, which is similar to that in the *cpeb1b* morphants, in the *cpeb1b* mutants injected with LD *cpeb1b* MO, but not in the wildtype (*SI Appendix*, Fig. S2 *M* and *N*), indicating that maternal effect indeed led to the alleviation of phenotype. Previous studies have demonstrated that HE specification is a prerequisite for HSPC generation during early development (2, 4, 8); therefore, we wondered whether HE specification was affected by Cpeb1b-deficiency. We observed the HE cells by using the Tg(*gfi1*:GFP) transgenic line and found that the number of *gfi1* positive HE cells within the VDA region was decreased in the *cpeb1b* morphants (Fig. 1 *I* and *J*). The flow cytometry analysis showed similar results (*SI Appendix*, Fig. S2 *O* and *P*). Furthermore, WISH showed that there were fewer HE cells in the *cpeb1b* morphants compared to control (Fig. 1 *K* and *L*), indicating the importance of Cpeb1b in HE specification. Taken together, these results suggest that Cpeb1b is required for HE specification, and Cpeb1b-deficiency–induced HE defect eventually leads to HSPC number reduction.

### Cpeb1b Regulates HSPC Development through Hh Signaling.

**Fig. 2.**
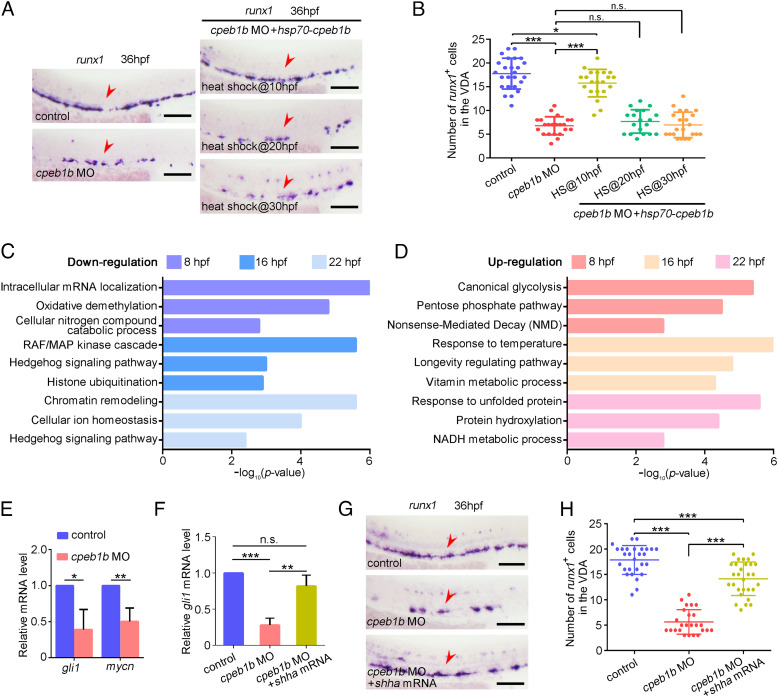
Cpeb1b regulates HSPC development through Hh signaling. (*A*) Examination of the *runx1* expression in the control, *cpeb1b* morphants, and Cpeb1b-overexpressed *cpeb1b* morphants by WISH. Cpeb1b overexpression was carried out by *hsp70*-*GFP*-*cpeb1b* HS at 10 hpf or 20 hpf or 30 hpf. The red arrowheads denote HSPCs. (Scale bar, 100 μm.) (*B*) Statistical analysis of the WISH. Error bar, mean ± SD, n.s.: no significance, *P < 0.05, ***P < 0.001. (*C*) The LD *cpeb1b* MO injected WT and *cpeb1b* mutant embryos at different developmental stages were collected for RNA-seq. The GO analysis showing the downregulated signaling pathways in *cpeb1b* mutant embryos. (*D*) GO analysis showing the upregulated signaling pathways in *cpeb1b* mutant embryos. (*E*) Relative mRNA level of Shh signaling downstream gene *gli1* and *mycn* in the control and *cpeb1b* morphants at 16 hpf examined by qRT-PCR. Error bar, mean ± SD. The *P* value was calculated by Student’s *t* test, *P < 0.05, **P < 0.01. (*F*) Relative mRNA level of *gli1* in the control, *cpeb1b* morphants, and *shha* mRNA-overexpressed *cpeb1b* morphants at 16 hpf examined by qRT-PCR. Error bar, mean ± SD. The *P* value was calculated by Student’s *t* test, n.s.: no significance, **P < 0.01, ***P < 0.001. (*G*) Examination of the *runx1* expression in the control, *cpeb1b* morphants, and *shha* mRNA-overexpressed *cpeb1b* morphants at 36 hpf by WISH. (Scale bar, 100 μm.) (*H*) Statistical analysis of the WISH. Error bar, mean ± SD, ***P < 0.001.

To further determine the timing of Cpeb1b function, we applied *hsp70* promoter-mediated heat-shock (HS) inducible Cpeb1b overexpression system. Strong green fluorescence of green fluorescent protein (GFP)-Cpeb1b was detected from 2 h post heat shock at indicated time points to at least 20 h post heat shock (*SI Appendix*, Fig. S3*A*). WISH showed that heat shock induction of Cpeb1b at 10 hpf, but not at 20- or 30 hpf, rescued the HSPC defect in the *cpeb1b* morphants (Fig. 2 *A* and *B*), suggesting that Cpeb1b functions in HSPC production at the somitic period. To investigate the mechanism upon Cpeb1b-deficiency, we collected WT and *cpeb1b* mutants at different developmental stages (8 hpf, 16 hpf, and 22 hpf) for RNA-seq analysis. Gene ontology (GO) analysis revealed that the downregulated and upregulated genes in Cpeb1b-deficient embryos were enriched in various signaling pathways, such as canonical glycolysis, mitogen-activated protein kinase cascade, Hh signaling pathway, histone ubiquitination, cellular ion homeostasis, vitamin metabolic process, and others (Fig. 2 *C* and *D* and Dataset S1). Among these altered signaling pathways in GO analysis, the Hh signaling, which was downregulated in Cpeb1b-deficient embryos at 16 and 22 hpf, has been previously demonstrated to regulate HSPC generation in zebrafish and mouse (10, 11). Combined with the finding that the Hh ligand and *cpeb1b* were highly coexpressed in the notochord, we speculated that Hh signaling might be involved in Cpeb1b deficiency-induced HSPC defects. qRT-PCR results showed that the expression of Hh signaling downstream genes *gli1* and *mycn* was reduced in the *cpeb1b* morphants (Fig. 2*E*). To determine whether Hh signaling induction can rescue the HSPC defect in *cpeb1b*-deficient embryos, we performed overexpression of *shha*, which is important for Hh signaling activation in zebrafish. The *shha* overexpression efficiency and its function on Hh signaling induction were confirmed (*SI Appendix*, Fig. S3 *B*–*D*). qRT-PCR showed that the reduced expression of Hh downstream gene *gli1* in the *cpeb1b* morphants was rescued by s*hha* overexpression (Fig. 2*F*), and WISH showed that the HSPC defect was also rescued (Fig. 2 *G* and *H*). Furthermore, we treated the *cpeb1b* morphants with the well-known Hh signaling agonist purmorphamine and found that purmorphamine treatment also provided a rescue effect (*SI Appendix*, Fig. S3 *E* and *F*). On the contrary, inhibition of the Hh signaling by its antagonist cyclopamine, which directly binds to and inhibits Hh signaling coreceptor and transducer Smo (39), impaired HSPC production (*SI Appendix*, Fig. S3 *G* and *H*), confirming a critical role of Hh signaling in HSPC development. In addition, we noticed that the Cpeb1b-deficiency–induced phenotype was weaker than that in embryos with cyclopamine treatment (*SI Appendix*, Fig. S3 *G* and *H*), implying that not all the Hh member-mediated signaling is inactivated in Cpeb1b-deficient embryos. Taken together, these results above indicate that Cpeb1b regulates HSPC development through Hh signaling.

Previous studies showed that Vegf signaling acts downstream of Hh signaling and upstream of the Notch signaling during early development, and this Hedgehog–Vegf–Notch signaling axis is crucial for HSPC generation (10, 40). Besides, Notch signaling is essential for HE specification, which is a prerequisite for HSPC generation (12, 13). To explore whether this signaling axis is involved in Cpeb1b-deficiency–induced HSPC defect, we performed the following assays. WISH showed that the expression of *vegfa* was decreased in the *cpeb1b* morphants compared with control (*SI Appendix*, Fig. S4 *A* and *B*), indicating that Cpeb1b-deficiency leads to downregulation of Vegf signaling. WB showed that the protein level of NICD was decreased in the *cpeb1b* morphants (*SI Appendix*, Fig. S4 *C* and *D*), and qRT-PCR showed that the expression of Notch signaling downstream genes *hey1* and *hey2* was also decreased in the *cpeb1b* morphants (*SI Appendix*, Fig. S4*E*). Next, we took advantage of the Notch reporter line Tg(*fli1a*:EGFP/*tp1*:mCherry) to examine Notch activity in ECs and found that the number of *fli1a*^+^*tp1*^+^ cells in the VDA region was reduced (*SI Appendix*, Fig. S4 *F* and *G*), demonstrating that Cpeb1b-deficiency resulted in attenuated Notch activity. Furthermore, WISH showed that the expression of Notch signaling-related artery genes *efnb2a* and *dll4* was also decreased in the *cpeb1b* morphants at 26 hpf (*SI Appendix*, Fig. S4 *H* and *I*). By contrast, the expression of another arterial marker *tbx20* was relatively normal in the *cpeb1b* morphants (*SI Appendix*, Fig. S4 *H* and *I*), suggesting that the dorsal aorta still developed in the Cpeb1b-deficient embryos, which is consistent with previous studies in that Notch signaling alteration does not necessarily result in loss of dorsal aorta (13, 41, 42). However, a complete inhibition of Hh signaling by cyclopamine led to nearly absent expression of *tbx20* in the trunk region (*SI Appendix*, Fig. S4 *J* and *K*), implying that the phenotype severity might be dependent on the Hh signaling level. To investigate whether Notch signaling activation can rescue the HSPC phenotype, we utilized a system for HS–inducible overexpression of NICD, which is a dominant activator of Notch signaling. The function of NICD overexpression on Notch signaling activation was confirmed (*SI Appendix*, Fig. S4*L*). qRT-PCR showed that the reduced expression of Notch downstream gene *hey2* in the *cpeb1b* morphants was rescued by NICD overexpression (*SI Appendix*, Fig. S4*M*), and WISH showed that the HSPC phenotype in the *cpeb1b* morphants was also rescued by NICD overexpression (*SI Appendix*, Fig. S4 *N* and *O*). Combined with the previous data, these results suggest that the Hedgehog–Vegf–Notch signaling axis is involved in Cpeb1b-mediated regulation of HSPC development.

### Cpeb1b Interacts with *shha* mRNA in the Liquid-Like Condensates.

**Fig. 3.**
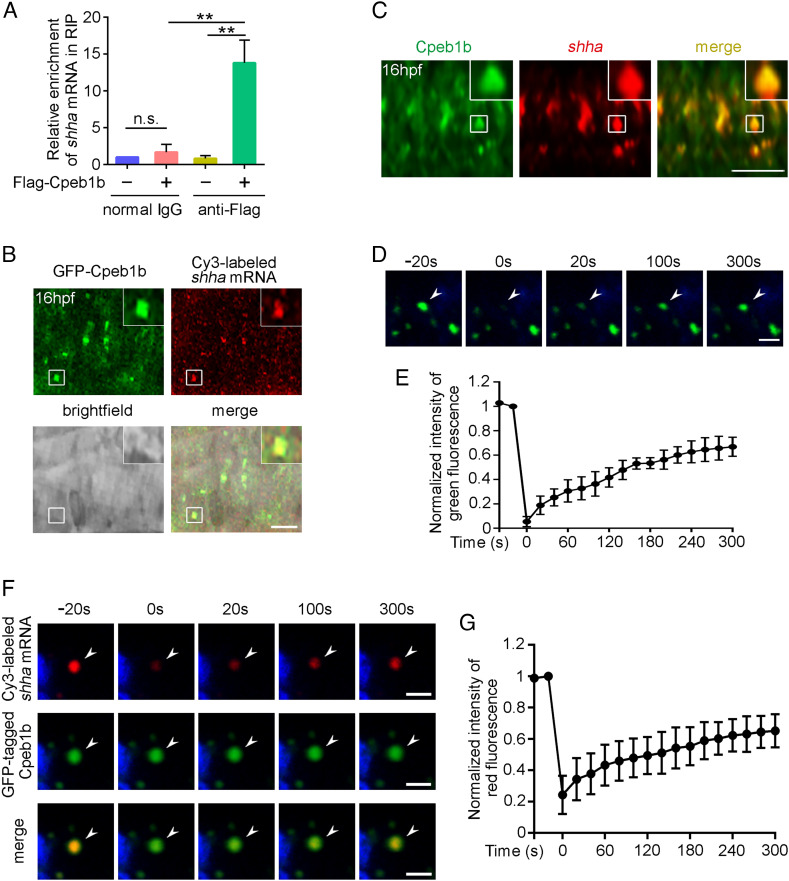
Cpeb1b interacts with *shha* mRNA in the liquid-like condensates of cytoplasm. (*A*) Relative mRNA level of *shha* in normal IgG and anti-Flag groups examined by qRT-PCR. Error bar, mean ± SD. The *P* value was calculated by Student’s *t* test, n.s.: no significance, **P < 0.01. (*B*) Confocal imaging shows colocalization of GFP-Cpeb1b and Cy3-labeled *shha* mRNA in the notochord of 16 hpf embryos. (Scale bar, 10 μm.) (*C*) The endogenous *shha* mRNA was detected by FISH, and the endogenous Cpeb1b was detected by IF using an antibody that can specifically recognize the antigen (444 to 468 amino acids) in Cpeb1b. (Scale bar, 10 μm.) (*D*) The representative images of fluorescence recovery of GFP-Cpeb1b droplets. (Scale bar, 2 μm.) (*E*) Relative quantification of fluorescence recovery kinetics of GFP-Cpeb1b droplets. Three condensates were tested, and three condensates were recovered. The lapsed time was 320 s. The black curve shows the mean ± SD (n = 3). (*F*) The representative images of fluorescence recovery of Cy3-labeled *shha* mRNA. (Scale bar, 2 μm.) (*G*) Relative quantification of fluorescence recovery kinetics of Cy3-labeled *shha* mRNA. Three condensates were tested, and three condensates recovered. The lapsed time was 320 s. The black curve shows the mean ± SD (n = 3).

To further explore the molecular mechanism of Cpeb1b in HSPC development, we performed RNA immunoprecipitation (RIP)-seq by using Flag-Cpeb1b to identify target mRNAs of Cpeb1b in embryos. Intriguingly, the result showed that Cpeb1b coimmunoprecipitated with *shha* (Dataset S2), which is important for Hh signaling induction (10, 40). A previous study has demonstrated that the definitive hematopoiesis is defective in the zebrafish *shha* mutant, *sonic-you* (*syu*) (10). Consistently, we knocked down Shha by injection of *shha* MO and found that the *runx1* expression was severely reduced in the *shha* morphants (*SI Appendix*, Fig. S5 *A* and *B*), which is similar to that in the *shha* mutant. Together with the observation that *cpeb1b* was colocalized with the *shha* in the notochord region of 16 hpf zebrafish embryo by FISH, we thus focused on *shha* mRNA for subsequent analysis. The interaction between Cpeb1b and *shha* mRNA in RIP-seq was further confirmed by RIP-qPCR assay (Fig. 3*A*). To observe the subcellular localization of Cpeb1b and *shha* mRNA, we cotransfected *GFP*-*cpeb1b* plasmid and Cy3-labeled *shha* mRNA into HEK293T cells. Confocal imaging showed that GFP-Cpeb1b colocalized with Cy3-labeled *shha* mRNA in a granular pattern in the cytoplasm of HEK293T cells (*SI Appendix*, Fig. S5*C*). To determine whether this also occurs in zebrafish embryo, we coexpressed GFP-Cpeb1b and Cy3-*shha* in embryos and found that GFP-Cpeb1b colocalized with Cy3-*shha* in a similar granular pattern in the notochord region of 16 hpf embryos (Fig. 3*B*). Furthermore, we detected the endogenous *shha* mRNA by FISH and utilized the immunofluorescence (IF) to detect the endogenous Cpeb1b. The confocal imaging showed similar result (Fig. 3*C*). Previous studies showed that condensation has different effects on movement and behavior of molecules (43–45), then we wondered whether the molecules in these Cpeb1b-positive condensates were trapped or dynamic. Fluorescence recovery after photobleaching (FRAP) assay was carried out in HEK293T cells by using a 488-nm laser, and the result showed recovery of GFP-Cpeb1b fluorescence within the bleached region (Fig. 3 *D* and *E* and Movie S2), suggesting that Cpeb1b molecules in these condensates could diffuse and exchange with surrounding dilute solution. Moreover, pairs of the GFP-Cpeb1b droplets could fuse and combine into one droplet (*SI Appendix*, Fig. S5*D* and Movie S3), further demonstrating liquid-like characteristics of these condensates. In addition, we performed FRAP on Cy3-*shha* by using a 561-nm laser and found recovery of Cy3-*shha* fluorescence within the bleached region (Fig. 3 *F* and *G* and Movie S4), suggesting that Cy3-*shha* in these condensates could also freely exchange with surrounding dilute solution. Taken together, these results above illustrate that Cpeb1b interacts with *shha* mRNA in the liquid-like condensates. Previous studies reported that some of the CPEB family members can undergo liquid–liquid phase separation by their prion-like domains (PLDs) (30, 46–48), then we wondered whether Cpeb1b also has this character. We analyzed the PLDs of zebrafish CPEB family members by using the prion-like amino acid composition (PLAAC) algorithm (49) and surprisingly found that there were no any PLDs within Cpeb1b, which was different from other CPEB family members (*SI Appendix*, Fig. S5*E*). Moreover, we performed in vitro cell-free phase separation assay by using purified Cpeb1b protein, which was labeled with Alexa488, and found that Cpeb1b did not form condensates (*SI Appendix*, Fig. S5*F*), indicating no phase separation ability of Cpeb1b itself. Considering that CPEB family proteins interact with poly(A)-binding protein (PABP) family proteins within cytoplasmic polyadenylation complex (24, 25), and many PABP family proteins have PLDs (50), we next asked whether formation of these condensates relies on the PABP family. Through searching the previous zebrafish gene expression pattern data, we found that *pabpc1b* was highly expressed in the notochord region during somitic period (51), which is consistent with *cpeb1b* expression. This finding was also confirmed by our WISH (*SI Appendix*, Fig. S6*A*). To reveal the subcellular localization of Pabpc1b, we cotransfected *mCherry*-*pabpc1b* and *GFP*-*cpeb1b* plasmids into HEK293T cells. Confocal imaging showed that mCherry-Pabpc1b colocalized with GFP-Cpeb1b in the cytoplasmic condensates (*SI Appendix*, Fig. S6*B*). Consistently, mCherry-Pabpc1b also colocalized with GFP-Cpeb1b in punctate pattern in the notochord region of 16 hpf embryos (*SI Appendix*, Fig. S6*C*). We analyzed the PLDs of Pabpc1b using the PLAAC algorithm and found that there indeed existed PLDs, which was similar to the ubiquitously expressed human homologous gene PABPC1 (*SI Appendix*, Fig. S6*D*). Then, we performed in vitro phase separation assay by using Alexa488-labeled Pabpc1b protein. The imaging showed that Pabpc1b formed spherical condensates in 150 mM but not 1 M KCl condition (*SI Appendix*, Fig. S6*E*), and FRAP showed recovery of Alexa488-Pabpc1b fluorescence within the bleached droplets (*SI Appendix*, Fig. S6 *F* and *G* and Movie S5). These results suggest that Pabpc1b was able to undergo phase separation, and the condensates formed by Pabpc1b were sensitive to high ionic strength, which can severely affect electrostatic interactions. To examine the intracellular Pabpc1b phase separation, we transfected *mCherry*-*pabpc1b* plasmid into HEK293T cells and found that Pabpc1b alone formed very few of condensates (*SI Appendix*, Fig. S6 *H* and *I*). By contrast, cotransfection of *pabpc1b* and *cpeb1b* led to much more condensates (*SI Appendix*, Fig. S6 *H* and *I*), implying that Cpeb1b might promote intracellular Pabpc1b phase separation. In addition, we knocked down Pabpc1b by *pabpc1b* MO injection and found that the *runx1* expression at 36 hpf was reduced in the *pabpc1b* morphants compared with control (*SI Appendix*, Fig. S6 *J* and *K*), suggesting that Pabpc1b is also important for HSPC production.

### The CPE Motif in *shha* mRNA Is Crucial for Cpeb1b Binding.

**Fig. 4.**
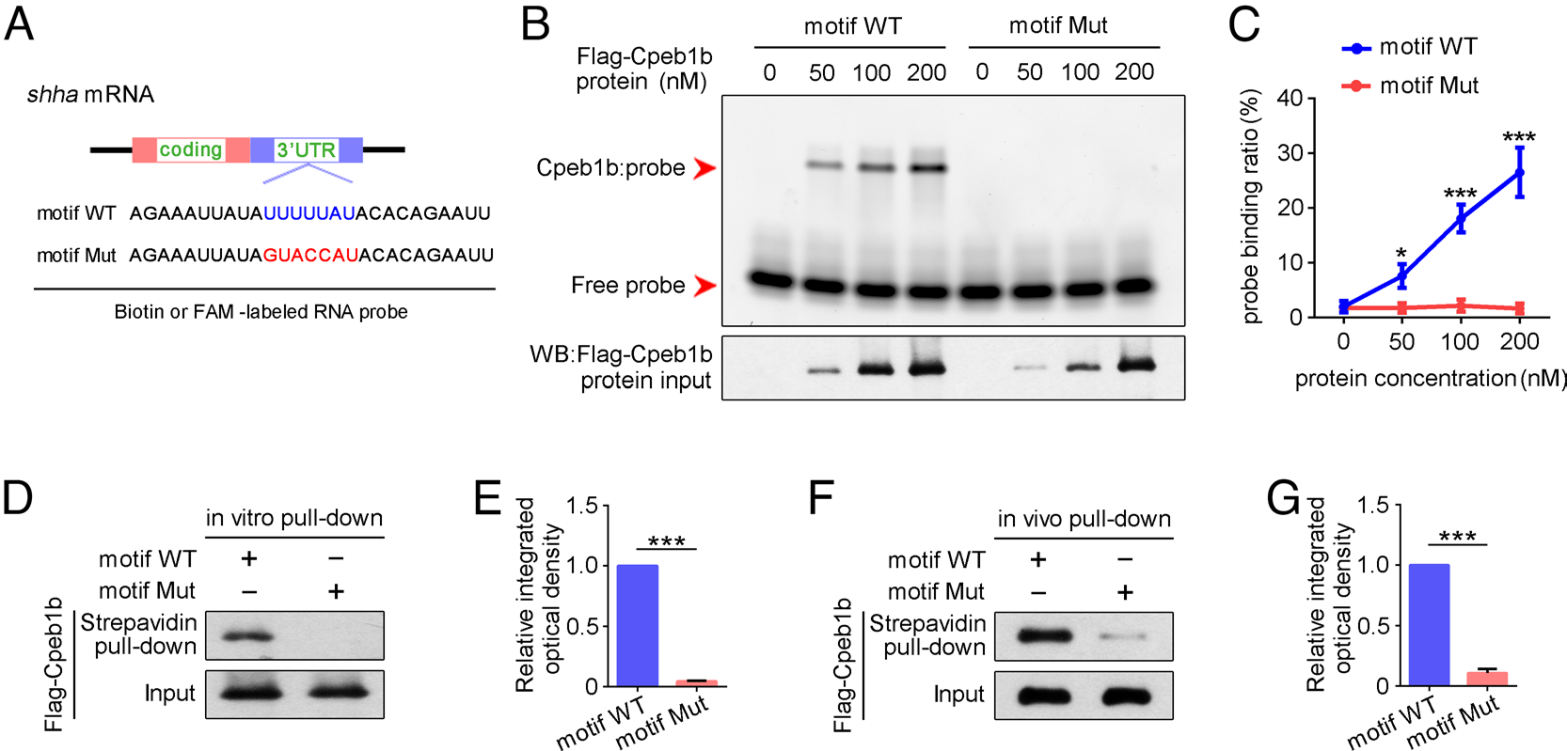
The CPEB motif in *shha* mRNA is important for Cpeb1b binding. (*A*) Diagram of biotin or FAM-labeled CPEB motif probe. The WT CPEB binding motif is in blue color, and the mutant CPEB binding motif is in red color. The sequence of motif WT and motif Mut probes is listed. (*B*) EMSA measuring the Cpeb1b binding with FAM-labeled motif WT and motif Mut probes. The protein concentration ranged from 0 nM to 200 nM. (*C*) Quantification of each band by gray analysis (Gel-Pro analyzer). The probe binding ratio at each group was determined by (RNA-protein)/[(free RNA) + (RNA-protein)]. GraphPad Prism was used for statistical analysis with Student’s *t* test. Statistic data were shown as mean ± SD. The *P* value was used for significance evaluation, *P < 0.05, ***P < 0.001. (*D*) WB showing the purified Flag-Cpeb1b protein pulled down with biotin-labeled CPEB motif probe. (*E*) Quantification of relative Flag-Cpeb1b protein level using gray analysis. Error bar, mean ± SD. The *P* value was calculated by Student’s *t* test, ***P < 0.001. (*F*) WB showing the Flag-Cpeb1b protein from *Flag*-*cpeb1b* mRNA-injected embryo extracts pulled down with biotin-labeled CPEB motif probe. (*G*) Quantification of relative Flag-Cpeb1b protein level using gray analysis. Error bar, mean ± SD. The *P* value was calculated by Student’s *t* test, ***P < 0.001.

Previous studies demonstrated that the specificity of CPEB protein binding relies on the 3′ UTR-located U-rich CPE motif (consensus sequence UUUUUAU) (24, 25), which was also found in *shha* mRNA (Fig. 4*A*), then we wondered whether this CPE motif is required for Cpeb1b binding. To examine whether Cpeb1b recognizes the CPE motif in 3′ UTR of *shha* mRNA, we designed RNA probes with WT and mutant CPE motif (Fig. 4*A*) and performed electrophoretic mobility shift assay (EMSA). The purified Flag-Cpeb1b protein was mixed with an FAM-labeled RNA probe that contained WT or mutant CPE motif, and the protein–probe complexes were analyzed on a non–denaturing polyacrylamide gel. The EMSA showed retardation of the WT motif probe but not the mutant probe (Fig. 4 *B* and *C*), indicating that CPE motif of *shha* is important for Cpeb1b binding. We performed in vitro streptavidin-biotin pull-down assay by using purified Flag-Cpeb1b protein and biotin-labeled probes and found that Flag-Cpeb1b was only pulled down by probe containing WT CPE motif (Fig. 4 *D* and *E*). Moreover, the in vivo pull-down assay was carried out by using the extracts from *Flag-cpeb1b* mRNA-injected embryos, and the result was consistent with the in vitro pull-down assay (Fig. 4 *F* and *G*), further confirming specific interaction between Cpeb1b and the CPE motif of *shha* mRNA.

### Cpeb1b Regulates Cytoplasmic Polyadenylation of *shha* mRNA.

**Fig. 5.**
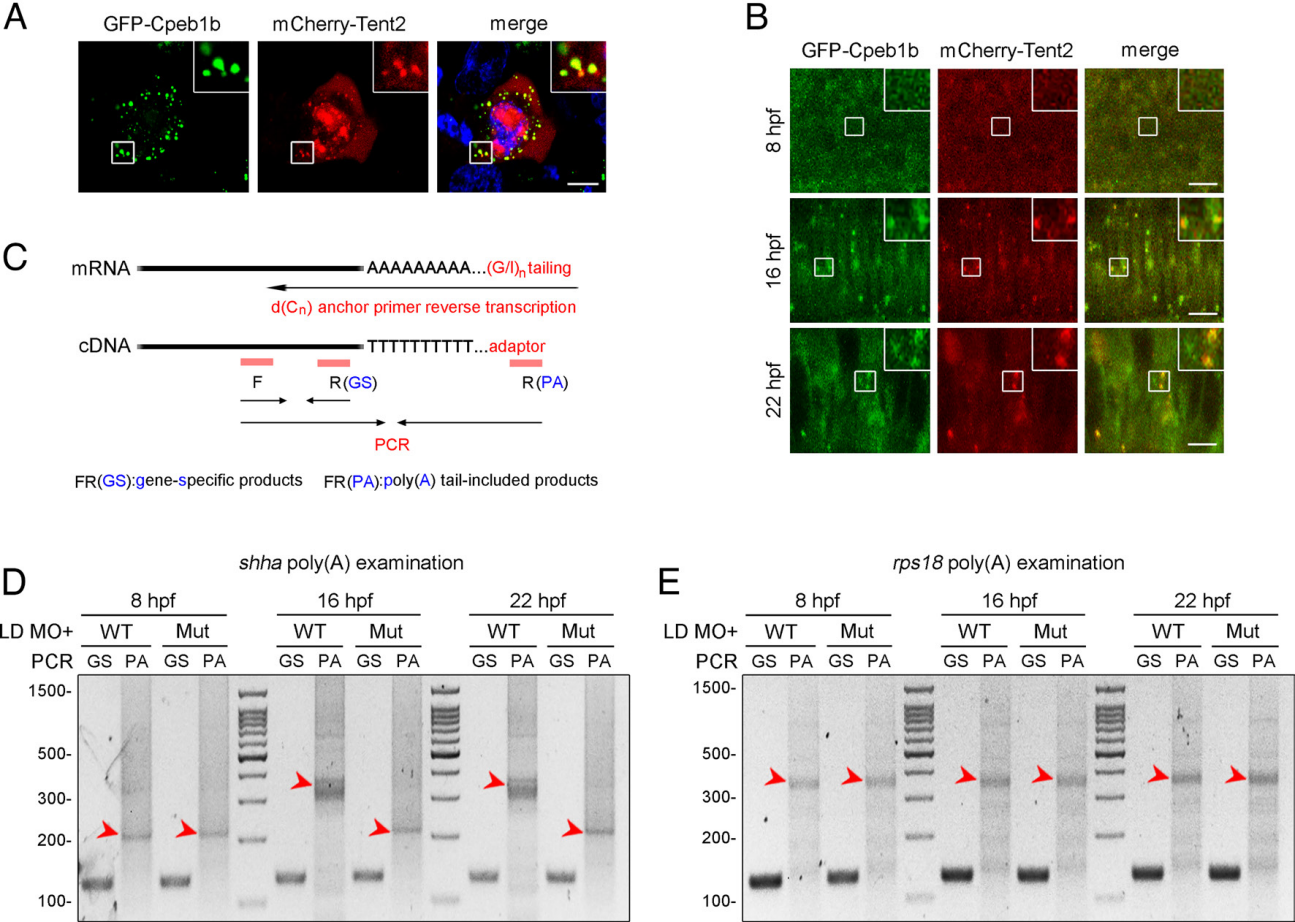
Cpeb1b regulates cytoplasmic polyadenylation of *shha* mRNA. (*A*) Confocal imaging shows colocalization of GFP-Cpeb1b and mCherry-Tent2 in the cytoplasm of HEK293T cells. (Scale bar, 10 μm.) (*B*) Confocal imaging shows that GFP-Cpeb1b colocalizes with mCherry-Tent2 in the condensates in the notochord of 16 and 22 hpf embryos but not in the hypoblast of 8 hpf embryos. (Scale bar, 10 μm.) (*C*) Outline of the PAT assay. Cytoplasmic RNA extracted from the embryos was subjected to G/I tailing. The complementary DNA was synthesized by reverse transcription using the poly(G/I) tailed RNA as template. Then, the specific PCR products were synthesized by specific primers and detected by gel electrophoresis. GS: gene-specific products, PA: poly(A) tail-included products. (*D*) Examination of the poly(A) length of *shha* mRNA in the LD *cpeb1b* MO-injected WT and *cpeb1b* mutant embryos (elimination of maternal effects) at different developmental stages by PAT assay. The PCR products were detected by gel electrophoresis. The red arrowheads denote smear bands from poly(A) PCR products. (*E*) Examination of the poly(A) length of *rps18* mRNA in the LD *cpeb1b* MO-injected WT and *cpeb1b* mutant embryos at different developmental stages. The red arrowheads denote smear bands from poly(A) PCR products.

In *Xenopus* and mammals, CPEB-regulated cytoplasmic polyadenylation is reported to rely on the CPEB partner Tent2, which as a noncanonical poly(A) polymerase is response for cytoplasmic poly(A) lengthening (26, 27). Based on these previous reports, we asked whether Cpeb1b interacts with Tent2 to facilitate cytoplasmic polyadenylation. We cotransfected *GFP*-*cpeb1b* and *mCherry*-*tent2* plasmids into HEK293T cells. Confocal imaging showed that GFP-Cpeb1b colocalized with mCherry-Tent2 in the cytoplasmic condensates (Fig. 5*A*), suggesting interaction between these two proteins. We further examined the localization of these proteins in embryos at different developmental stages and observed that GFP-Cpeb1b colocalized with mCherry-Tent2 in the condensates in the notochord of 16 and 22 hpf embryos, but not in the hypoblast of 8 hpf embryos (Fig. 5*B*). To further address whether Cpeb1b, Pabpc1b, Tent2, and *shha* mRNA form a complex together, we utilized a multicolor fluorescent labeling system. The confocal imaging showed the colocalization of GFP-Cpeb1b, mCherry-Pabpc1b, BFP-Tent2, and Cy5-*shha* mRNA in the cytoplasmic condensates of HEK293T cells (*SI Appendix*, Fig. S7*A*), suggesting that these molecules can form a complex together. Then, we examined the poly(A) length of *shha* mRNA in embryos at different developmental stages by PCR poly(A) test (PAT) assay (Fig. 5*C*) (52). Gel electrophoresis showed that the poly(A) length of *shha* at 8 hpf was shorter than that at 16 or 22 hpf, and the poly(A) length between 16 and 22 hpf was similar (*SI Appendix*, Fig. S7*B*), suggesting an important poly(A) length shift between 8 and 16 hpf. Next, we investigated the Cpeb1b-regulated cytoplasmic polyadenylation. The PAT assay revealed that the poly(A) tail length of *shha* mRNA in the *cpeb1b* mutants was decreased at 16 and 22 hpf, but not at 8 hpf, compared with that in the WT (Fig. 5*D*). By contrast, there was no difference in the poly(A) tail length of *rps18* mRNA, which was not the Cpeb1b target transcript, between the WT and mutant embryos at different developmental stages (Fig. 5*E*). Taken together, these results indicate that Cpeb1b regulates cytoplasmic polyadenylation of *shha* mRNA.

### Cpeb1b Facilitates Shha Protein Expression through Translational Control.

**Fig. 6.**
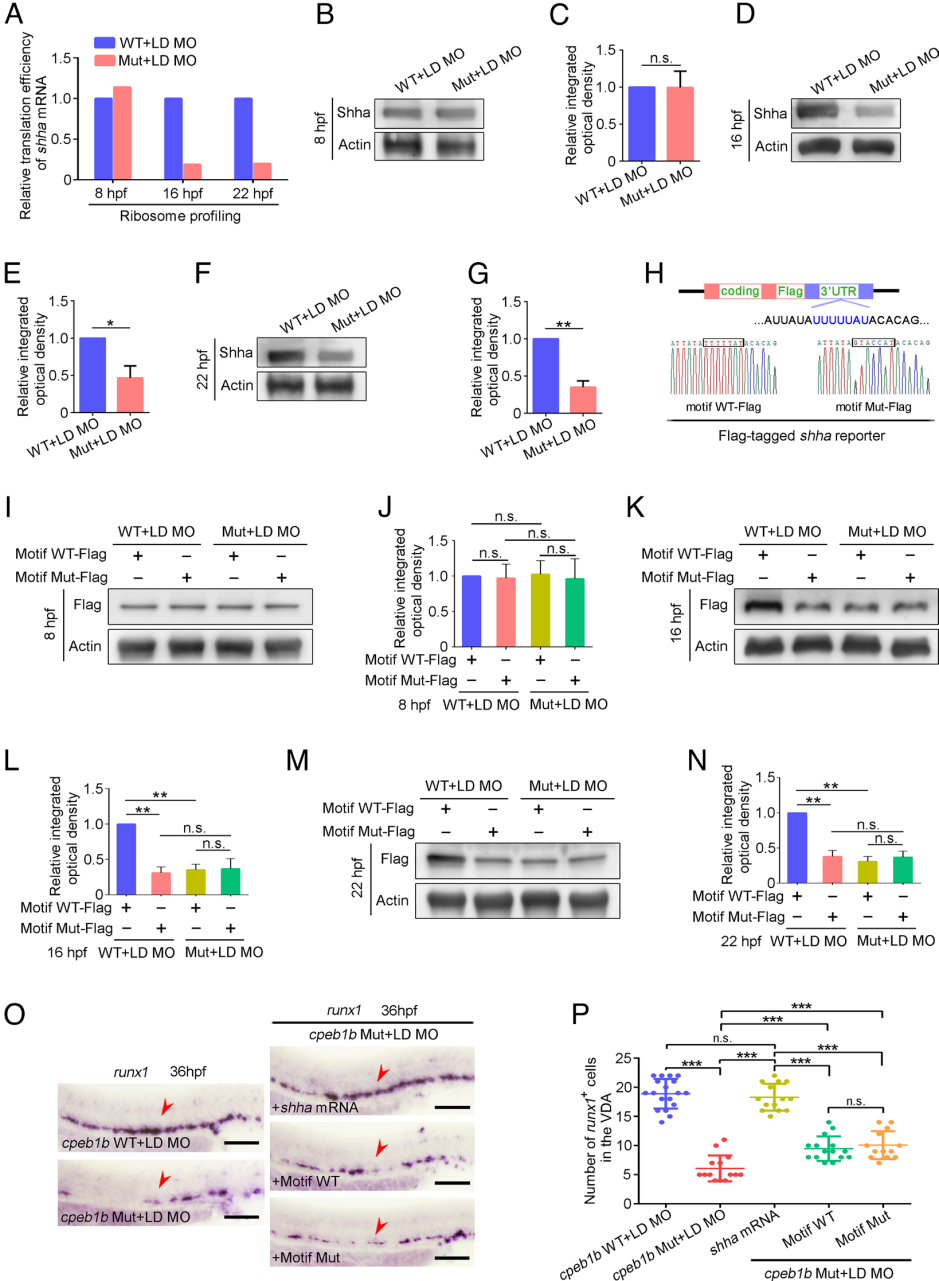
Cpeb1b regulates translation of *shha* mRNA. (*A*) Relative translation efficiency of *shha* mRNA in the LD *cpeb1b* MO injected WT and *cpeb1b* mutant embryos examined by ribosome profiling. (*B*) Protein level of endogenous Shha and Actin in the LD *cpeb1b* MO-injected WT and *cpeb1b* mutant embryos at 8 hpf examined by WB. (*C*) Quantification of relative Shha protein level using gray analysis. Two replicates, error bar, mean ± SD. The *P* value was calculated by Student’s *t* test, n.s.: no significance. (*D*) Protein level of endogenous Shha and Actin in the LD *cpeb1b* MO-injected WT and *cpeb1b* mutant embryos at 16 hpf examined by WB. (*E*) Quantification of relative Shha protein level using gray analysis. Two replicates, error bar, mean ± SD. The *P* value was calculated by Student’s *t* test, **P < 0.01. (*F*) Protein level of endogenous Shha and Actin in the LD *cpeb1b* MO-injected WT and *cpeb1b* mutant embryos at 22 hpf examined by WB. (*G*) Quantification of relative Shha protein level using gray analysis. Two replicates, error bar, mean ± SD. The *P* value was calculated by Student’s *t* test, *P < 0.05. (*H*) Diagram of Flag-tagged *shha* reporter. The CPEB binding motif is in blue color, and partial sequence of the motif WT and motif Mut reporters is listed. (*I*) Protein level of Shha-Flag and Actin in Motif WT-Flag or Motif Mut-Flag overexpressed WT and *cpeb1b* mutant embryos at 8 hpf examined by WB. Elimination of maternal effects was carried out by the LD *cpeb1b* MO injection. (*J*) Quantification of relative Shha-Flag protein level using gray analysis. Two replicates, error bar, mean ± SD. The *P* value was calculated by Student’s *t* test, n.s.: no significance. (*K*) Protein level of Shha-Flag and Actin in Motif WT-Flag or Motif Mut-Flag overexpressed WT and *cpeb1b* mutant embryos at 16 hpf examined by WB. (*L*) Quantification of relative Shha-Flag protein level using gray analysis. Two replicates, error bar, mean ± SD. The *P* value was calculated by Student’s *t* test, n.s.: no significance, **P < 0.01. (*M*) Protein level of Shha-Flag and Actin in Motif WT-Flag or Motif Mut-Flag overexpressed WT and *cpeb1b* mutant embryos at 22 hpf examined by WB. (*N*) Quantification of relative Shha-Flag protein level using gray analysis. Two replicates, error bar, mean ± SD. The *P* value was calculated by Student’s *t* test, n.s.: no significance, **P < 0.01. (*O*) Examination of the expression of *runx1* by WISH. The red arrowheads denote HSPCs. LD: a low dose of *cpeb1b* MO injection. In HSPC phenotype rescue experiments using *cpeb1b* mutant embryos, these embryos were obtained by cross-mating *cpeb1b* adult mutants and individually subjected to genotyping after WISH experiment for identifying homozygous mutants. (Scale bar, 100 μm.) (*P*) Statistical analysis of the WISH. Error bar, mean ± SD, n.s.: no significance, ***P < 0.001.

Previous studies showed that polyadenylation selectively regulates mRNA stability or translation and further affects protein level in different biological processes (20), then we asked whether Cpeb1b-mediated cytoplasmic polyadenylation is involved in regulation of mRNA metabolism and protein level during HSPC development. We first examined the expression level of *shha* mRNA in the control and *cpeb1b* morphants at 16 hpf by WISH and surprisingly found no difference between the two groups (*SI Appendix*, Fig. S7 *C* and *D*). To examine the stability of *shha* mRNA, we treated the embryos with α-amanitin for inhibition of polymerase II-mediated nascent mRNA transcription and detected the relative level of *shha* mRNA in the control and *cpeb1b* morphants at the indicated time intervals by qRT-PCR. The 18s rRNA, which is transcribed by polymerase I and not sensitive to α-amanitin, was used as an internal control. The qRT-PCR result revealed that Cpeb1b deficiency did not alter the stability of *shha* mRNA (*SI Appendix*, Fig. S7*E*). Next, to explore whether the translational efficiency of *shha* mRNA was affected, we performed ribosome profiling assays and found that the global and *shha* mRNA translational efficiency in the *cpeb1b* mutants was decreased at 16 and 22 hpf, but not at 8 hpf (Fig. 6*A*, *SI Appendix*, Fig. S7 *F*–*H*, and Dataset S3). Consistently, WB analysis showed a decreased endogenous Shha protein level (Fig. 6 *B*–*G*). Furthermore, to determine whether the *shha* mRNA translation is CPE motif-dependent, we designed *shha*-*Flag* reporter mRNAs with WT and mutant CPE motif (Fig. 6*H*). After validating the reporter mRNA stability (*SI Appendix*, Fig. S7*I*), we showed that in WT embryos, the Shha-Flag expression was reduced in mutant CPE motif groups at 16 and 22 hpf, but not at 8 hpf, compared with that in the WT CPE motif groups (Fig. 6 *I*–*N*). However, in the *cpeb1b* mutant embryos, there was no difference between the two groups that contained different reporters (Fig. 6 *I*–*N*). In addition, we synthesized another Cpeb1b nontarget and polyadenylated *shha* mRNA as control. This mRNA, whose 3′UTR is from SV40 Poly(A) terminator, was in vitro polyadenylated. We respectively overexpressed these mRNAs in the *cpeb1b* mutant embryos and found that the polyadenylated *shha* mRNA almost fully rescued the HSPC defect (Fig. 6 *O* and *P*). However, the CPE motif WT and motif Mut mRNA had only a little but similar rescue effect (Fig. 6 *O* and *P*). Collectively, these results indicate that Cpeb1b regulates the Shha protein level though translational control of *shha* mRNA.

### Cpeb1b Deficiency Mildly Affects the Development of Nervous System and Somite, but Not Pectoral Fin.

In consideration of the role of Hh signaling is broad and not restricted to HSPC development (10, 53), we performed additional experiments to explore whether Cpeb1b-deficiency affects other organs and systems. WISH showed that the expression of muscle cell marker *myod* (54, 55) at 18 and 24 hpf was slightly reduced in the *cpeb1b* morphants compared with control (*SI Appendix*, Fig. S8 *A* and *B*), suggesting a mild somite developmental defect induced by Cpeb1b-deficiency. In addition, the *fkd4*, which is another downstream gene of Hh signaling (54, 56), expression in the hindbrain and ventral neural tube of *cpeb1b* morphants was also slightly reduced (*SI Appendix*, Fig. S8 *C* and *D*), suggesting a mild nervous system developmental defect. However, the pectoral fin development, which is also regulated by Hh signaling (54), was normal in the *cpeb1b* morphants (*SI Appendix*, Fig. S8 *E* and *F*).

## Discussion

**Fig. 7.**
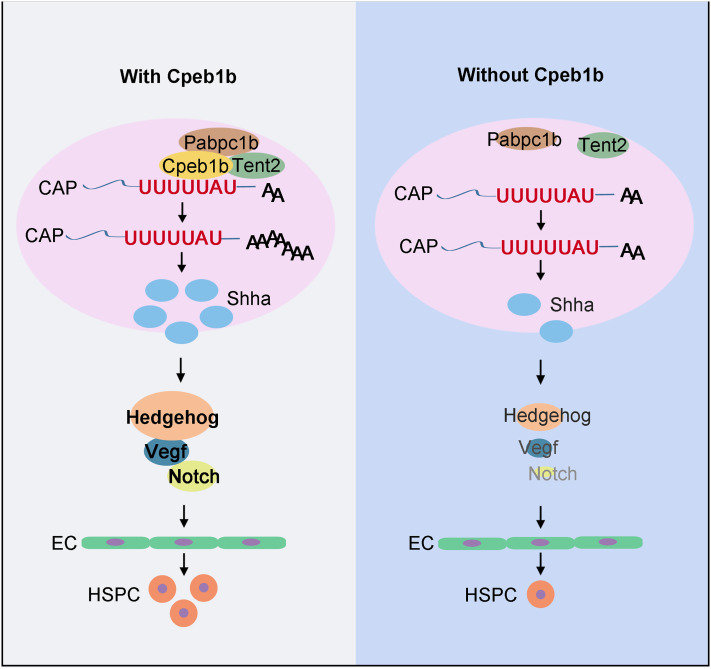
Schematic diagram showing that Cpeb1b modulates HSPCs development via cytoplasmic polyadenylation of *shha* mRNA. Cpeb1b-mediated cytoplasmic polyadenylation of *shha* mRNA is important for enhancing its translation efficiency and therefore increases the Shha protein level. An increase in the number of Shha protein is required for definitive hematopoiesis by regulating Hedgehog–Vegf–Notch signaling axis. In the absence of Cpeb1b, the Shha protein level is reduced, which results in downregulation of Hedgehog–Vegf–Notch signaling axis, further leading to definitive hematopoiesis defect.

During HSPC development, how the signals are elaborately regulated to ensure the in vivo cell fate transition is a critical biological question. In this study, we have investigated the role of Cpeb1b in HSPC production and demonstrated the function of cytoplasmic polyadenylation in this developmental process in the zebrafish model. We find that the cytoplasmic polyadenylation regulator Cpeb1b is highly expressed in the notochord region during somitic period and is required for HSPC emergence. The Cpeb1b-deficiency leads to downregulation of the Hedgehog–Vegf–Notch signaling axis, further resulting in HE specification defect. Mechanistically, Cpeb1b interacts with *shha* mRNA through the CPE motif in the liquid-like condensates, and Cpeb1b-deficiency inhibits cytoplasmic polyadenylation of *shha* mRNA, further impairing its translation. Altogether, these findings suggest that Cpeb1b-mediated cytoplasmic polyadenylation plays an important role in HSPC development through translational control of Hh signaling (Fig. 7).

In vertebrates, Hh signaling, as a crucial developmental signal, is regulated by various factors to improve the signal transduction accuracy. At the transcriptional level, HNF3β transcription factor binds to the specific HNF3β motif in *shh* promoter and activates *shh* transcription, and the misexpression of HNF3β results in *shh* ectopic expression (57–59). Moreover, the retinoic acid receptors and retinoid X receptors heterodimers bind to the RA response element in *shh* promoter and can respond to retinoic acid (RA) stimulation, further inducing RA-dependent *shh* transcription (59, 60). At the posttranslational level, the covalent modification by cholesterol at the N terminus of Hh protein is essential for restricting its spatial deployment, subsequently affecting cellular response (61). Besides, palmitoylation modification at the N terminus of Hh protein, which is promoted by the ski/HHAT acyl transferase, facilitates membrane association and secretion of Hh ligand (62). In addition to these transcriptional and posttranslational regulations of Hh signaling, we discover in this HSPC developmental study that Cpeb1b-mediated cytoplasmic polyadenylation may supply another layer of posttranscriptional regulation of Hh signaling. We show that 1) the expression of *cpeb1b* is enriched in notochord region during somitic period and colocalizes with *shha*, which contributes to spatial and temporal specificity at the tissue level; 2) the interaction between Cpeb1b and *shha* mRNA is dependent on the particular CPE motif and is important for regulating translation but not stability of *shha* mRNA, contributing to specificity at the molecular level. Although there are many other mRNAs binding to Cpeb1b in the RIP-seq data, based on a series of experiments on phenotype and signaling pathway analysis, we speculate that the *shha* should be the main and direct target responsible for the HSPC defect in the Cpeb1b-deficient embryos. Moreover, multiple signals besides Hh were altered in Cpeb1b-deficient embryos in our GO analysis. The signals, such as canonical glycolysis, histone ubiquitination, cellular ion homeostasis, response to unfolded protein, and vitamin metabolic process, are involved in physiological-biological activities of glucose homeostasis, histone modification, ion transport, unfolded protein response, and vitamin metabolism regulation. Whether these signals are indeed regulated by Cpeb1b as well as being involved in HSPC development awaits further investigation.

In addition, we show that Cpeb1b-deficiency mildly affects the development of nervous system and somite, which is known to be regulated by Hh signaling (54–56), suggesting that the role of Cpeb1b is not strictly restricted to HSPC development, and Cpeb1b-deficiency–induced HSPC defect is part of a more widespread developmental defects in different organs. Surprisingly, we find that the pectoral fin development, which is also reported to be regulated by Hh signaling (54), is not affected by Cpeb1b-deficiency. In consideration of the inactivation of Hh signaling in Cpeb1b-deficient embryos is incomplete and the development of different tissues depends on distinct levels of Hh signaling activity (53, 63), we speculate that zebrafish HSPC production may be more sensitive to Hh signaling alteration than pectoral fin development and then could be affected by Cpeb1b-deficiency more easily.

Biomolecular condensates as important cellular biochemistry organizers can not only influence fundamental properties of biomolecules but also provide membrane-less compartments for precise biochemical reaction, which may contribute to ensuring accuracy of signal transduction (43–45). Previous studies report that some proteins of CPEB family have the prion-like characters and can undergo aggregation, which is nonpathological but critical for their physiological functions. In *Aplysia*, the multimerization of ApCPEB, which is induced by its N-terminal PLD, is enhanced by serotonin stimulation and is essential for the maintenance of long-term facilitation by upregulation of local protein synthesis in neurons (46, 64). Similarly, the oligomerization of *Drosophila* CPEB Orb2 is critical for its function in translational control and persistence of long-term memory (30, 47). In addition, the PLD-mediated aggregation of mouse CPEB3 promotes the translation of AMPA receptors and facilitates persistence of memory (65). In this study, we show that Cpeb1b colocalizes with *shha* mRNA and poly(A) polymerase Tent2 in the cytoplasmic condensates under normal conditions. Based on the results of FRAP and granule fusion assays, we find that the molecules in these condensates are dynamic, suggesting liquid-like but not pathological or gel-like characteristics of these condensates. Intriguingly, Cpeb1b does not have PLDs and cannot undergo aggregation by itself, which is different from other CPEB members. Nevertheless, Cpeb1b colocalizes with Pabpc1b and efficiently promotes Pabpc1b’s phase separation ability, which further induces the condensate formation. Although this condensation phenomenon in Cpeb1b-mediated cytoplasmic polyadenylation is evident, the detailed molecular mechanism remains elusive. At the subcellular level, condensation changes the spatial distribution and local quantity of biomolecules (43–45, 66). We speculate that the condensation in cytoplasmic polyadenylation may increase the local concentration of specific enzymes and mRNAs, further improving the accuracy and efficiency of catalytic reaction. However, more studies are awaited to demonstrate this point.

Previous study showed that in Hodgkin’s lymphoma-derived cell line, the CPEB1-coordinated alternative polyadenylation and splicing with translational regulation are involved in regulation of genes important for hematologic malignancy progression (67). Moreover, the CPEB1 expression was reported to be significantly reduced in multiple myeloma cells (68), implying that CPEB1 may be related to myeloma progression. However, the role of CPEB1 in normal hematopoiesis in animal models has not been elucidated thus far. Our study shows that Cpeb1b-mediated cytoplasmic polyadenylation is important for zebrafish HSPC production through translational control during early embryogenesis. The appropriate cytoplasmic polyadenylation of *shha* mRNA facilitates its own translation in the notochord region and therefore activates the Hh signaling during the critical phase of HSPC development. These findings may provide some clues to answer how developmental signals are precisely controlled by posttranscriptional regulation and direct ECs to execute fate transition to HSPCs.

## Materials and Methods

The information of ethics statement, zebrafish husbandry, MOs, vector construction, mRNA synthesis, microinjection, WISH, double FISH, generation of mutant by Crispr/Cas9, quantitative RT-PCR, WB, cell culture and transfection, protein purification, in vitro condensates formation assay, microscopy imaging, FRAP assay, EMSA, in vitro RNA pulldown assay, in vivo RNA pulldown assay, PAT assay, RNA-seq, GO analysis, RIP experiment, and ribosome profiling experiment are in *SI Appendix*.

## Supplementary Material

Appendix 01 (PDF)

Dataset S01 (XLSX)

Dataset S02 (XLSX)

Dataset S03 (XLSX)

Movie S1. **The blood flow in the control and Cpeb1b-deficient embroys.** Time-lapse imaging was performed to observe the blood flow in the control and *Cpeb1b* morphants. Scale bar, 100 μm.

Movie S2. **Fluorescence signal recovery of GFP-Cpeb1b droplet.** Time-lapse imaging was performed to observe the fluorescence signal recovery of GFP-Cpeb1b droplet in HEK293T cells. A bleaching 488 nm laser pulse was applied. In this representative movie, one condensate was tested, and one condensate recovered. The lapsed time was 420 s. Scale bar, 10 μm.

Movie S3. **The fusion of GFP-Cpeb1b droplet.** Time-lapse imaging was performed to observe the fusion of GFP-Cpeb1b droplet in HEK293T cells. In this representative movie, twenty condensates were showed, and four condensates fused. The lapsed time was 180 s. Scale bar, 2 μm.

Movie S4. **Fluorescence signal recovery of Cy3-labeled *shha* mRNA.** Time-lapse imaging was performed to observe the fluorescence signal recovery of Cy3-labeled *shha* mRNA in HEK293T cells. A bleaching 561 nm laser pulse was applied. In this representative movie, one condensate was tested, and one condensate recovered. The lapsed time was 420 s. Scale bar, 10 μm.

Movie S5. **Fluorescence signal recovery of Alexa488-labeled Pabpc1b in vitro.** Time-lapse imaging was performed to observe the in vitro fluorescence signal recovery of Alexa488-labeled Pabpc1b. A bleaching 488 nm laser pulse was applied. In this representative movie, one condensate was tested, and one condensate recovered. The lapsed time was 420 s. Scale bar, 2 μm.

## Data Availability

The RNA-seq, RIP-seq, and ribosome profiling sequencing data supporting the conclusions of this article have been deposited in the Gene Expression Omnibus database (GEO: GSE207906).
