# Supplementary material for: Cpeb1b-mediated cytoplasmic polyadenylation of shha mRNA modulates zebrafish definitive hematopoiesis
Source: Proc Natl Acad Sci U S A. 2023 Feb 6;120(7):e2212212120. doi: 10.1073/pnas.2212212120 (PMC9964029; doi:10.1073/pnas.2212212120)
Supplement: Supplementary file 1 — Appendix 01 (PDF) [file pnas.2212212120.sapp.pdf]

Supplementary Information for

Cpeb1b-mediated cytoplasmic polyadenylation of *shha* mRNA modulates zebrafish definitive hematopoiesis

Jian Heng[a,b,1,2], Boyang Shi[c,1], Jia-Yi Zhou[c,d,1], Yifan Zhang[a,b,d], Dongyuan Ma[a,b], Yun-Gui Yang[c,d,2], Feng Liu[a,b,d,e,2]

[a] State Key Laboratory of Membrane Biology, Institute of Zoology, Chinese Academy of Sciences, Beijing, China; [b] Institute for Stem Cell and Regeneration, Chinese Academy of Sciences, Beijing, China; [c] CAS Key Laboratory of Genomic and Precision Medicine, Collaborative Innovation Center of Genetics and Development, College of Future Technology, Beijing Institute of Genomics, Chinese Academy of Sciences and China National Center for Bioinformation, Beijing, China; [d] University of Chinese Academy of Sciences, Beijing, China; [e] School of Life Sciences, Shandong University, Qingdao, 266237, China

[1] J.H., B.S., and J.Z. contributed equally to this work.
[2] To whom correspondence may be addressed. Email: liuf@ioz.ac.cn; ygyang@big.ac.cn; hengjian@ioz.ac.cn

SI Appendix

SI Experimental Procedures

Ethics Statement. Our zebrafish experiments were all approved and carried out in accordance with the Animal Care Committee at the Institute of Zoology, Chinese Academy of Sciences (permission number: IOZ-IACUC-2021-017).

Zebrafish Husbandry and Chemical Treatment. Zebrafish strains including AB, Tubingen, *Tg*(*flk1*:mCherry) (1), *Tg*(*fli1a*:EGFP) (2), *Tg*(*cmyb*:GFP) (3), *Tg*(*tp1*:mCherry) (4), *Tg*(*gfi1*:GFP) (5) were raised in system water at 28.5°C under standard conditions. The zebrafish embryos were got by natural spawning. For cyclopamine treatment, 100 μM cyclopamine (Sigma) was used in medium to treat embryos from 10 hpf. For purmorphamine treatment, 25 μM purmorphamine (Sigma) was used in medium to treat embryos from 10 hpf. For α-amanitin treatment, 0.4 ng α-amanitin (Sigma) was injected into the yolk region of 14 hpf embryos.

Morpholinos, Vector Construction, mRNA Synthesis and Microinjection. Morpholino oligonucleotides (MOs) used in this study, including:

cpeb1b MO: 5'-CCATGGCAGGTCAAACAACCAAAAT-3' (Gene Tools)

shha MO: 5'-CAGCACTCTCGTCAAAAGCCGCATT-3' (a gift from Dr. Luo) (6)

pabpc1b MO: 5'-GGGTTCATTTTCTCCTCAGTCGCAG-3' (Gene Tools)

control MO: 5'-CCTCTTACCTCAGTTACAATTTATA-3' (Gene Tools)

MOs were injected into one-cell stage embryos (6 ng for *cpeb1b* MO, 3.2 ng for *shha* MO, 6 ng for *pabpc1b* MO, 3.2-6 ng for control MO, 2 ng for low-dose of *cpeb1b* MO). For vector construction, Flag-tagged or GFP-tagged *cpeb1b* CDS (with or without *cpeb1b* MO binding site), Flag-tagged *shha* CDS, mCherry-tagged *pabpc1b* CDS, mCherry or BFP-tagged *tent2*, CDS were respectively cloned into pCS2^{+} vector. For mRNAs synthesis and injection, mRNAs were generated using SP6 mMessage Machine kit (Invitrogen) and injected into one-cell stage embryos. For HSPC rescue experiment, the 3'UTR in the *shha* mRNA was from SV40 polyA terminator, and this mRNA was in vitro polyadenylated by using Poly(A) Tailing Kit (Ambion). The 3'UTR in *shha* CPE motif-WT or motif-Mut mRNA was from *shha* gene, and these mRNAs were not in

vitro polyadenylated. For mRNA co-localization assay, the T7 promoter sequence-fused PCR product of the *shha* mRNA was used as template for in vitro transcription. Cy3- or Cy5-labeled mRNA was generated using the MEGAscript™ T7 Transcription Kit (Invitrogen) with Cy3-UTP (Enzo Life Sciences) or Cy5-UTP (Enzo Life Sciences). For observation of GFP-Cpeb1b and Cy3 labeled *shha* mRNA in embryos, we co-microinjected the GFP-Cpeb1b and Cy3 labeled *shha* mRNA into one-cell stage embryos for their overexpression. And for observation of GFP-Cpeb1b and mCherry-Pabpc1b in embryos, we co-microinjected the GFP-Cpeb1b and mCherry-Pabpc1b into one-cell stage embryos. For heat-shock induction of GFP-Cpeb1b overexpression, GFP-tagged *cpeb1b* CDS (without *cpeb1b* MO binding site) was cloned into pHSP vector with the *hsp70* promoter. For highly efficient overexpression, the plasmid was co-injected with Tol2 mRNA into the one-cell stage embryos as previously described (7). The heat-shock condition was 30 min at 42 °C.

Whole-mount in situ Hybridization and Double Fluorescence in situ Hybridization. Whole-mount in situ hybridization was carried out using Digoxigenin-uridine-5'-triphosphate (Roche) labeled RNA probes. For probes synthesis, gene specific PCR products were cloned into pGEM-T vector (Promega). The RNA probe was transcribed with T7 RNA polymerase (Promega). After hybridization, RNA probes were detected by AP-conjugated anti-DIG antibody (Roche) using BM purple (Roche) as substrate. For double fluorescence in situ hybridization assay, one probe was labeled with DIG and detected by POD-conjugated anti-DIG antibody and the other probe was labeled with fluorescein and detected by POD-conjugated anti-fluorescein antibody. The TSA Plus Cy3 Solution (PerkinElmer) and TSA Plus Flourescein Solution (PerkinElmer) were used as substrate respectively.

Generation of Mutant by CRISPR/Cas9 and Identification of Mutant. The *cpeb1b* mutant was generated using CRISPR/Cas9 technology. Linearized pXT7-Cas9 plasmid was used as template for in vitro transcription of *Cas9* mRNA. The *Cas9* mRNA was generated using T7 mMessage Machine kit (Invitrogen) and treated with DNase I (NEB). Then *Cas9* mRNA was purified using RNA clean Kit (TIANGEN). The T7 promoter sequence-fused PCR product of the gRNA scaffold

plasmid was used as template for in vitro transcription of gRNA. The *cpeb1b* gRNA, with the target sequence 5'-GGAGACTAGTGGCTTCAGCTC-3', was generated by in vitro transcription by T7 RNA polymerase (Promega) and was treated with DNase I (NEB) for degradation of the DNA template. The gRNA was purified using mirVana™ miRNA Isolation Kit (Ambion). Then the *Cas9* mRNA and gRNA were co-injected into one-cell stage embryos for genome editing. The mutant identification was carried out by high resolution melting curve (HRM) and DNA sequencing analysis. The *cpeb1b* zygotic mutants could grow up to adulthood; however, the defective germ cell and ovary in females transformed themselves into males, and then no homozygous adult female could be obtained. In HSPC phenotype rescue experiments using *cpeb1b* mutant embryos, the low-dose *cpeb1b* MO injected embryos, which were obtained by cross-mating *cpeb1b* adult mutants, were individually lysed (lysis buffer: 10 mM Tris-HCl pH 8.0, 2 mM EDTA, 0.1% Triton X-100, 200 μg/ml Proteinase K; lysis condition: 55°C for 3 h, and then 95°C for 10 min) and subjected to genotyping after WISH experiment for identifying homozygous mutants. The primers used for genotyping in this study are listed as follows:

cpeb1b mut identification-F: 5'-GGACTCCCAGTCAGCTTAC-3'

cpeb1b mut identification-R: 5'-CAAGAGTGGAGGGACCAGAC-3'

Quantitative RT-PCR. Quantitative reverse-transcription PCR (qRT-PCR) was carried out to examine the relative abundance of target RNAs. 2 μg of total RNA extracted from zebrafish embryos was used for cDNA synthesis using the M-MLV Reverse Transcriptase (Promega). Experiments were performed according to the qPCR manufacturer's instructions (TIANGEN) and examined by the CFX96 Real-Time PCR System (Bio-Rad). Experiments were performed at least three biological replicates. GraphPad Prism was used for statistical analysis with Student's t-test. Statistic data were shown as mean ± S.D.. *P* value was used for significance evaluation. n.s.: no significance, *$P<0.05$; **$P<0.01$; ***$P<0.001$. The primers used for qRT-PCR in this study are listed as follows:

gapdh-F: 5'-GTCTTCACTACTATTGAGAAGGC-3'

gapdh-R: 5'-GCAGTTGGTGGTGCAGGAGGCA-3'

hey1-F: 5'-TCGAAGTGGAGAAGGAGAGTG-3'

hey1-R: 5'-TCCCTCCGGCGCTTCTCGAT-3'

hey2-F: 5'-GAAGCGGCCCTGTGAGGACA-3'

hey2-R: 5'-CTTTCTGGCCATGACTTGGGA-3'

gli1-F: 5'-TGTCCTGGGCAGTGTAATGGAC-3'

gli1-R: 5'-ATGGCCTCGGGTTCGGGTTTCTC-3'

mycn-F: 5'-AGACACGACAGTCAAAGCGCC-3'

mycn-R: 5'-GTCTCGCCTGCACTGACTTGAG-3'.

shha-F: 5'-TTCGGTTGCTGCGAAATCTGG-3'

shha-R: 5'-ACGTCGCGTCGTGGAGTCTCGG-3'

18s rRNA-F: 5'-ATTCGTATTGCGCCGCTAGAGG-3'

18s rRNA-R: 5'-ATCGCGGGTCGGCATCGTTTAC-3'

Western Blot and Immunofluorescence. The embryos were collected and lysed in lysis buffer (50 mM Tris-HCl pH 7.4, 150 mM NaCl, 1 mM EDTA, 0.5 mM DTT, 0.5% NP-40, 0.2% SDS, 5%(v/v) Glycerol, Roche cocktail protease inhibitor). The lysate was separated through SDS-PAGE, and the immunoblot was performed using the following antibodies: anti-β-Actin antibody (Cell Signaling Technology, 4967), anti-NICD antibody (Abcam, ab83232), anti-Flag (Sigma-Aldrich, F7425), anti-Shha (abcepta, Azb10041a). Gray analysis (Gel-Pro analyzer) was used for quantification of protein level. GraphPad Prism was used for statistical analysis with Student's t-test. Statistic data were shown as mean ± S.D.. *P* value was used for significance evaluation. n.s.: no significance, *P<0.05; **P<0.01; ***P<0.001. Immunofluorescence experiments were performed as previously reported (8) using the following antibodies: anti-Cpeb1 (abcepta, AP16631b).

Cell Culture and Transfection. Human HEK-293T cells were maintained in DMEM (Gibco) supplemented with 10% FBS (Gibco), 100 U/mL penicillin (Gibco) and 100 mg/mL streptomycin (Gibco) at humidified 37 °C, 5% CO_2 cell culture incubator. The plasmids or Cy3-labeled mRNA were transfected into cells using lipofectamine 3000 (Invitrogen) according to the manufacturer's instructions.

Protein Purification. The HEK293T cells were transfected with plasmids (Flag-tagged target gene) using the PEI transfection reagent (MKbio). After 48 h, the cells were lysed with lysis buffer (50 mM HEPES-KOH, pH 7.4, 1 M KCl, 1% NP-40, 1 mM DTT, 1 mM EDTA, protease inhibitor cocktail, benzonase) and sonicated (10% output, 10 s pulse-on, 20 s pulse-off) for 1 min by a Sonic Dismembrator (Thermo Fisher). After removing cell debris through centrifugation at 13,300 rpm for 10 min, the lysates were incubated with the anti-Flag M2 Affinity Gel (Sigma-Aldrich) for 4 h, at 4 °C. Samples were then washed five times with the lysis buffer and five times with TBS buffer. The gel-bound proteins were eluted with 4 mg/mL 3×Flag peptide (Sigma-Aldrich) by incubating the mixture for 1 h, at 4 °C. The eluate containing purified protein was concentrated using VIVASPIN 500 (Sartorius Stadium Biotech) and quantified by coomassie brilliant blue staining and BCA Protein Assay Kit (Thermo Fisher).

In vitro Condensates Formation Assay. The in vitro phase separation assay was performed as previously described (9, 10), with some modifications. Briefly, proteins were labeled with Alexa Fluor 488 NHS ester (Thermo Fisher) and were concentrated by ultrafiltration. The unlabeled protein solutions were diluted to the indicated concentrations. Then the Alexa488-labeled and unlabeled proteins were mixed at a molar ratio of 1:9 and incubated in the buffer (5 μM protein final concentration, 20 mM Tris-HCl pH 7.0, 10% PEG 3350, indicated salt concentration) at 25 °C. For ionic strength condition, we chose 150 mM KCl to mimic physiological ionic strength and 1 M KCl to provide high ionic strength, respectively. Samples were imaged with the Nikon A1 confocal microscopy.

Microscopy Imaging and FRAP Assay. High-resolution brightfield and fluorescent images were taken with the Nikon A1 confocal microscope as previously described (11). Ordinary brightfield and fluorescence photographs were imaged by the Nikon SMZ1500 microscope with a Nikon digital camera. FRAP experiments were carried out by the Nikon A1 confocal microscope. To analyze the condensates in HEK293T cells, a circular region was selected for FRAP assay. To analyze condensates in solution, a circular region containing one droplet was assayed. The

GFP-tagged or Alexa-488 labeled proteins were photobleached using the 488-nm laser beam at 50-80% power. The Cy3-labeled mRNA was photobleached using the 561-nm laser beam at 80% power. For quantitative analysis, the average fluorescence intensity of frames before photobleaching was normalized to 100%. Statistic data were analyzed using GraphPad Prism and were shown as mean ± S.D..

Electrophoretic Mobility Shift Assay (EMSA). The high concentrations of purified Flag-Cpeb1b proteins were diluted in binding buffer (50 mM Tris-HCl pH 7.4, 150 mM NaCl, 0.5 mM EDTA, 0.1% NP-40, 0.5 mM DTT, 5% glycerol, 5 ng/μl BSA, RNase inhibitor). The purified protein (0 nM, 50 nM, 100 nM, and 200 nM final concentration, respectively) and synthesized FAM-labeled RNA probes (100 nM final concentration) were mixed and incubated at room temperature for 30 min. Then, the glutaraldehyde (0.1% final concentration) was added into the mixture and incubated at room temperature for 15 min. After adding Hi-Density TBE sample buffer (Thermo Fisher), the samples were separated by 6% TBE gel electrophoresis for 30-60 min at 80 V. The gel was scanned by the Typhoon 9400 (GE Healthcare) imager. Quantification of each band was carried out by gray analysis (Gel-Pro analyzer). The RNA binding ratio at each group was determined by (RNA-protein)/((free RNA) + (RNA-protein)). GraphPad Prism was used for statistical analysis with Student's t-test. Statistic data were shown as mean ± S.D.. *P* value was used for significance evaluation. n.s.: no significance, *$P<0.05$; **$P<0.01$; ***$P<0.001$. The RNA probes used for EMSA assays are listed as follows:

motif WT: AGAAAUUAUAUUUUUAUACACAGAAUU-FAM

motif Mut: AGAAAUUAUAGUACCAUACACAGAAUU-FAM

In vitro Pulldown Assay. The biotin-labeled RNA probes were heated in metal-free water for 5 min at 65 °C, then flash-cooled on ice. Then, the RNA probes and purified Flag-Cpeb1b protein (from HEK293T cells transfected with *Flag-cpeb1b* plasmid) were incubated with streptavidin-conjugated magnetic beads (NEB), which were pre-cleared by incubation with 0.2 mg/ml tRNA (Sigma) and 0.2 mg/ml BSA (Amresco) for 1 h at 4 °C under gentle rotation, in binding buffer (50 mM Tris-HCl pH 7.4, 150 mM NaCl, 0.5 mM EDTA, 0.1% NP-40, 1 mM DTT,

protease inhibitor cocktail, RNase inhibitor) for 1 h at 4 °C. After washing with binding buffer for five times and with TBS buffer for five times, the bead-bound proteins were heated in NuPAGE™ LDS Sample Buffer (Invitrogen) and then separated by the SDS-PAGE electrophoresis, and were subjected to western blot analysis with anti-Flag antibody (Sigma-Aldrich). The RNA probes used for in vitro RNA pulldown assays are listed as follows:

motif WT: Biotin-AGAAAUUAUAUUUUUAUACACAGAAUU

motif Mut: Biotin-AGAAAUUAUAGUACCAUACACAGAAUU

In vivo Pulldown Assay. The in vivo pull-down assays were carried out using extracts from zebrafish embryos. *Flag-cpeb1b* mRNA-injected embryo extracts were precleared for 1 h at 4 °C by incubation with streptavidin-conjugated magnetic beads (NEB) in binding buffer (50 mM Tris-HCl pH 7.4, 150 mM NaCl, 0.5 mM EDTA, 0.1% NP-40, 1 mM DTT, protease inhibitor cocktail, RNase inhibitor). The biotin-labeled RNA probes were heated in metal-free water for 5 min at 65 °C, then flash-cooled on ice. The probes were incubated with pre-cleared zebrafish embryo extracts for 2 h at 4 °C under gentle rotation together with streptavidin-conjugated magnetic beads (NEB) which were pre-cleared by incubation with 0.2 mg/ml tRNA (Sigma) and 0.2 mg/ml BSA (Amresco) for 1 h at 4°C under gentle rotation. After washing with binding buffer for five times and with TBS buffer for five times, the bead-bound proteins were heated in NuPAGETM LDS Sample Buffer (Invitrogen) and then separated by the SDS-PAGE electrophoresis, and were subjected to western blot analysis with anti-Flag antibody (Sigma-Aldrich). The RNA probes used for in vivo RNA pulldown assays are listed as follows:

motif WT: Biotin-AGAAAUUAUAUUUUUAUACACAGAAUU

motif Mut: Biotin-AGAAAUUAUAGUACCAUACACAGAAUU

PCR Poly(A) Test (PAT) Assay. The PAT assays were carried out based on the protocol of Poly(A) Tail-Length Assay Kit (Thermo Fisher) and previous study (12), but with some modifications. Briefly, the zebrafish embryos were lysed in hypotonic lysis buffer (20 mM Tris-HCl pH 7.4, 10 mM KCl, 1 mM EDTA, 0.2% NP-40, RNase inhibitor) under vortex. After centrifugation, the supernatant was used for cytoplasmic RNA extraction. The cytoplasmic RNA was extracted from

the supernatant by TRIzol reagent (Invitrogen). Then the 2 μg cytoplasmic RNA was subjected to G/I tailing by the Tail Buffer Mix (Thermo Fisher) and Tail Enzyme Mix (Thermo Fisher) at 37°C for 90 min. After tailing reaction, the Tail Stop Solution (Thermo Fisher) was added to the mixture. The cDNA was synthesized by reverse transcription using the poly(G/I) tailed RNA as template. Then the specific PCR products were synthesized using HotStart-IT® Taq DNA Polymerase (Thermo Fisher) and specific primers. The PCR products were detected by gel electrophoresis. The primers used for PAT assays are listed as follows:

shha gene-specific-F: 5'-GAGTAACTACTGTACGTTTAC-3'

shha gene-specific-R: 5'-AGGCAGGTTAACTGGTATGG-3'

rps18 gene-specific-F: 5'-AGAGGCTGAAGAAGATCAGG-3'

rps18 gene-specific-R: 5'-CCTTTATTTGTAAATTTACTTC-3'

RNA-seq and Gene Ontology Analysis. The entire embryo was used for RNA-seq. The RNA-seq was performed in duplicates, and each group had 10 embryos per replicate. Total RNA was extracted from the embryos at different developmental stages by TRIzol reagent (Invitrogen) and the mRNA was purified using RiboMinus Eukaryote Kit v2 (Invitrogen). Fragmented mRNA was used for library construction using the KAPA RNA HyperPrep Kit (Kapa Biosystems). The quality of raw sequencing reads was screened by using FastQC. Low-quality bases were trimmed and filtered by cutadapt (V 1.13) (13) and Trimmomatic (V 0.36) (14). Processed reads were mapped to the *Danio rerio* (Zv9) genome information from the Ensembl annotation using hisat2 (V 2.0.5) (15) with default parameters. After quality filtering (≥ 20) by SAM tools (V1.9) (16), read counts and corresponding RPKMs were calculated. The fold change of expression level of transcripts between samples was calculated using the DEseq2 (V 1.26.0) package (17). Gene ontology (GO) analysis was performed using the Metascape (http://metascape.org/) (18).

RIP Experiment. *Flag-cpeb1b* mRNA injected embryos were collected at 16 hpf and lysed in NETN lysis buffer (150 mM NaCl, 0.5% NP-40, 50 mM Tris-HCl, pH 7.4, RNase inhibitor, Roche cocktail protease inhibitor). Cpeb1b RIP was carried out following the published protocol (19), but with some modifications. Briefly, the lysate was incubated with anti-FLAG M2 Magnetic Beads

(Merck) for 4 h at 4 °C. Then, the beads were washed five times with the lysis buffer and five times with TBS buffer. For RIP-qPCR assay, the bead-bound RNA was extracted and then reverse transcribed. The cDNA was subjected to qPCR assay. For RIP-seq assay, the bead-bound RNA was extracted and then fragmented by RNA Fragmentation Reagent (Ambion). The fragmented RNA was subjected to library construction using the SMARTer smRNA-Seq kit (Clontech). The quality of raw sequencing reads was screened by using FastQC and low-quality bases were trimmed and filtered by cutadapt (V 1.13) (13) and Trimmomatic (V 0.36) (14). Processed reads were mapped to the zebrafish genome (Zv9) using TopHat (v2.1.1) (20) with ''–bowtie1''. The target genes of Cpeb1b were identified using the MACS2 software (version 2.0.10) (21), and annotated based on Ensembl gene annotation information, using BEDTools' intersectBed (22).

Ribosome Profiling Experiment. Ribosome profiling experiments were performed as previously reported (23), with some modifications. Briefly, the zebrafish embryos were incubated in 100 mg/ml cycloheximide (Selleck) buffer (5 mM NaCl, 0.2 mM KCl, 0.3 mM $CaCl_2$, 0.3 mM $MgSO_4$) for 5 min at 28°C. The embryos were then transferred into ice-cold lysis buffer. After triturating the embryos through the needle, the lysate was clarified by recovering the soluble supernatant after centrifugation for 10 min at 20,000 g at 4°C. 3 μl of RNase I (100 U/μl) was added to 600 μl of lysate. After a 45 min incubation at room temperature with gentle mixing, 10 μl of SUPERase·In RNase inhibitor (Invitrogen) was added to stop the nuclease digestion. MicroSpin S-400 HR columns (GE Healthcare) were equilibrated with 3 ml of polysome buffer by gravity flow and emptied by centrifugation at 600 g for 4 min. Immediately 100 μl of the digested lysate was loaded on the column and eluted by centrifugation at 600 g for 2 min. RNA was extracted from the flow-through using Trizol reagent (Invitrogen). Ribosomal RNA fragments were removed using the RiboZero Kit (Illumina) and were separated on a 17% denaturing urea-PAGE gel (National Diagnostics). RNA fragments sized 27 nt to 30 nt were cut out from the gel, and were subjected to library preparation using the Smarter smRNA-Seq kit (Clontech). Single read sequencing data was used to obtain ribosome protected fragments RNA-seq data. The quality of raw sequencing reads was screened by using FastQC. Low-quality bases were trimmed and filtered by cutadapt (V 1.13) (13) and Trimmomatic (V 0.36) (14). Processed reads were mapped to the zebrafish rRNA transcriptome using Bowtie (24) and unmapped reads were retained. The

remaining reads were mapped to zebrafish genome (Zv9) using TopHat (v2.1.1) (20) with ‘‘–bowtie1’’. The expression levels of mRNAs were evaluated by Cufflinks v2.2.1 (25) with ‘‘-p x -u -G’’. Translational efficiency of each mRNA was calculated by the ratio of RPFs and expression of input mRNA.

Supplemental Dataset Legends

Dataset S1. The GO analysis of transcripts in the Cpeb1b-deficient embryos at different developmental stages.

Dataset S2. The enrichment of Cpeb1b target genes in RNA immunoprecipitation.

Dataset S3. The translation efficiency of transcripts in the WT and *cpeb1b* mutant embryos.

Supplemental Movie Legends

Movie S1. The blood flow in the control and Cpeb1b-deficient embryos. Time-lapse imaging was performed to observe the blood flow in the control and *cpeb1b* morphants. Scale bar, 100 μm.

Movie S2. Fluorescence signal recovery of GFP-Cpeb1b droplet. Time-lapse imaging was performed to observe the fluorescence signal recovery of GFP-Cpeb1b droplet in HEK293T cells. A bleaching 488 nm laser pulse was applied. In this representative movie, one condensate was tested, and one condensate recovered. The lapsed time was 420 s. Scale bar, 10 μm.

Movie S3. The fusion of GFP-Cpeb1b droplet. Time-lapse imaging was performed to observe the fusion of GFP-Cpeb1b droplet in HEK293T cells. In this representative movie, twenty condensates were showed, and four condensates fused. The lapsed time was 180 s. Scale bar, 2 μm.

Movie S4. Fluorescence signal recovery of Cy3-labeled *shha* mRNA. Time-lapse imaging was performed to observe the fluorescence signal recovery of Cy3-labeled *shha* mRNA in HEK293T

cells. A bleaching 561 nm laser pulse was applied. In this representative movie, one condensate was tested, and one condensate recovered. The lapsed time was 420 s. Scale bar, 10 μm.

Movie S5. Fluorescence signal recovery of Alexa488-labeled Pabpc1b in vitro. Time-lapse imaging was performed to observe the in vitro fluorescence signal recovery of Alexa488-labeled Pabpc1b. A bleaching 488 nm laser pulse was applied. In this representative movie, one condensate was tested, and one condensate recovered. The lapsed time was 420 s. Scale bar, 2 μm.

Supplemental Figures:

Figure S1

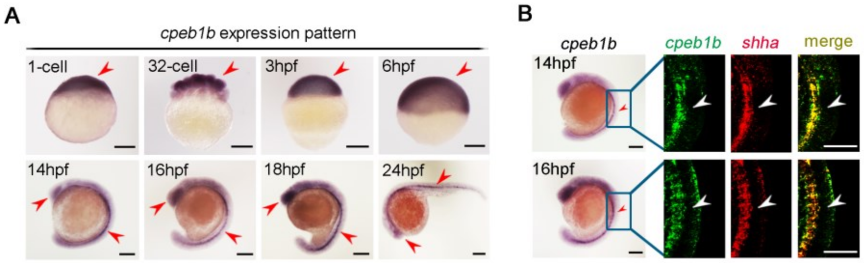

Fig. S1. The expression pattern of *cpeb1b* mRNA. (*A*) Expression pattern of *cpeb1b* mRNA examined by whole mount in situ hybridization (WISH). Scale bar, 200 μm. (*B*) The double fluorescence in situ hybridization (FISH) showed that the *cpeb1b* signals were colocalized with *shha* signals. The arrowheads denote notochord. Scale bar, 200 μm.

Figure S2

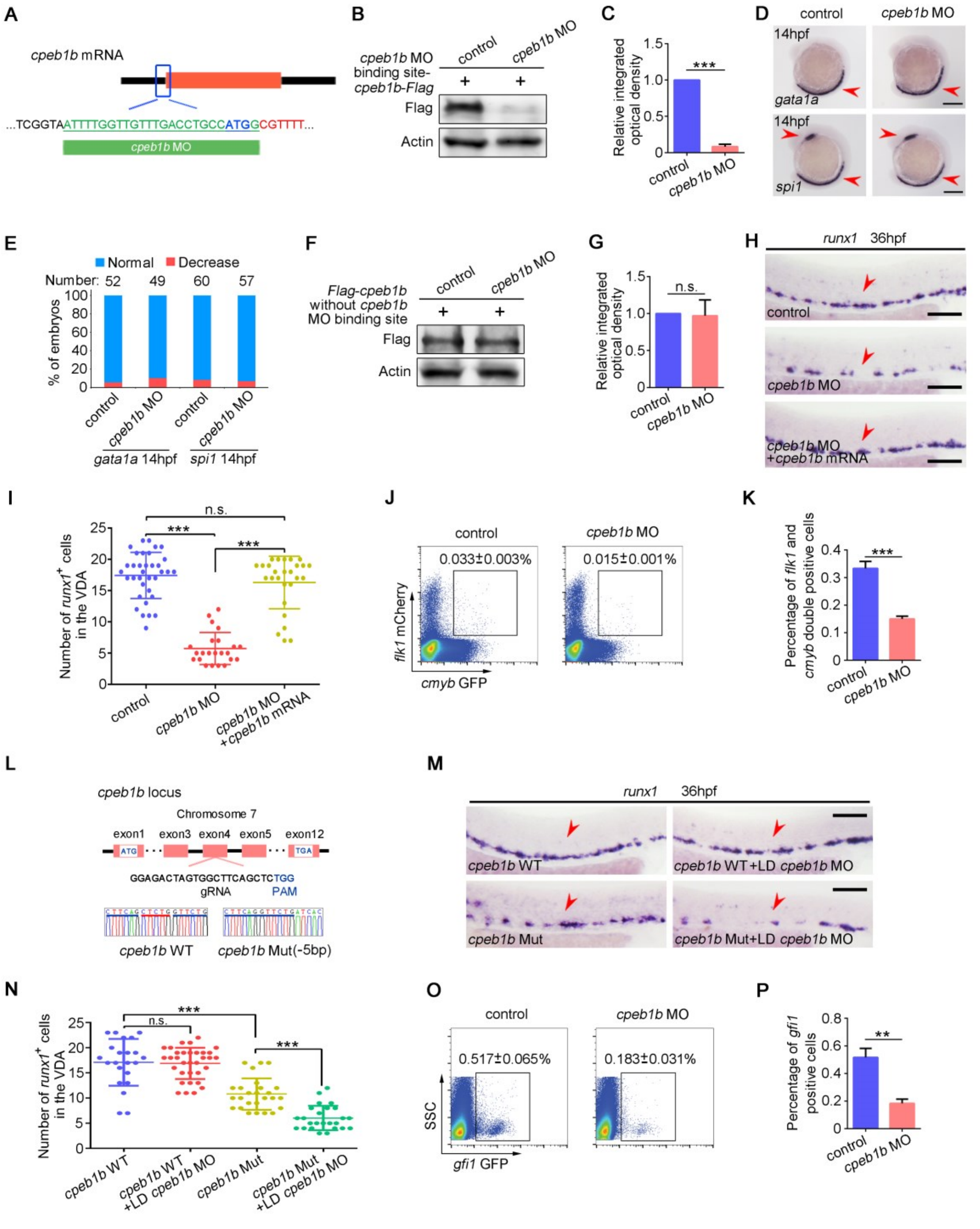

Fig. S2. Cpeb1b is important for definitive hematopoiesis but not primitive hematopoiesis. (*A*) The design of *cpeb1b* MO targeting the *cpeb1b* mRNA for blocking the translation. The *cpeb1b* MO target sequence is listed and the start codon is in blue color. (*B*) Knockdown efficiency of *cpeb1b* MO examined by western blot (WB). *Flag*-tagged mRNA containing both of the *cpeb1b* MO binding site and *cpeb1b* CDS was co-injected with either control MO or *cpeb1b* MO into one-cell stage embryos. The protein level of Cpeb1b-Flag and Actin was examined by WB. (*C*)

Quantification of relative Cpeb1b-Flag protein level using gray analysis (Gel-Pro analyzer). Error bar, mean ± S.D., *P* value was calculated by Student's t-test, ***$P < 0.001$. (*D*) Examination of erythroid marker *gata1a* and myeloid marker *spi1* at 14 hpf by WISH. (*E*) Quantification of the WISH. (*F*) The *Flag-cpeb1b* mRNA without *cpeb1b* MO binding site was co-injected with either control MO or *cpeb1b* MO into one-cell stage embryos. The protein level of Flag-Cpeb1b and Actin was examined by WB. (*G*) Quantification of relative Flag-Cpeb1b protein level using gray analysis. Error bar, mean ± S.D., *P* value was calculated by Student's t-test, n.s.: no significance. (*H*) WISH shows that *Flag-cpeb1b* mRNA without *cpeb1b* MO binding site can rescue the expression of HSPC marker *runx1* in the *cpeb1b* morphants. The red arrowheads denote HSPCs. Scale bar, 100 μm. (*I*) Statistical analysis of the WISH. Error bar, mean ± S.D., *P* value was calculated by Student's t-test, n.s.: no significance, ***$P < 0.001$. (*J*) Flow cytometry analysis of *flk1*$^+$*cmyb*$^+$ definitive hematopoietic precursors in the control and *cpeb1b* morphants. The trunk region tissue from the control and *cpeb1b* morphants (*flk1*:mCherry/*cmyb*:GFP background) was dissected and dissociated into single cells for Flow cytometry analysis. (*K*) The statistical analysis of flow cytometry analysis. Three replicates, error bar, mean ± S.D., *P* value was calculated by Student's t-test, ***$P < 0.001$. (*L*) Generation of *cpeb1b* 5 bp deletion mutant using CRISPR/Cas9. Location and sequence of the sgRNA target site are exhibited, and the partial genome sequence of the WT and *cpeb1b* mutant is listed. (*M*) Examination of *runx1* expression in a low dose (LD) of *cpeb1b* MO-injected WT and *cpeb1b* mutant embryos by WISH. (*N*) Statistical analysis of the WISH. Error bar, mean ± S.D., *P* value was calculated by Student's t-test, n.s.: no significance, ***$P < 0.001$. (*O*) Flow cytometry analysis of *gfi1*$^+$ hemogenic endothelium cells in the control and *cpeb1b* morphants. The trunk region tissue from the control and *cpeb1b* morphants (*gfi1*:GFP background) was dissected and dissociated into single cells for flow cytometry analysis. SSC gating was used for excluding cell debris. (*P*) The statistical analysis of flow cytometry analysis. Three replicates, error bar, mean ± S.D., *P* value was calculated by Student's t-test, **$P < 0.01$.

Figure S3

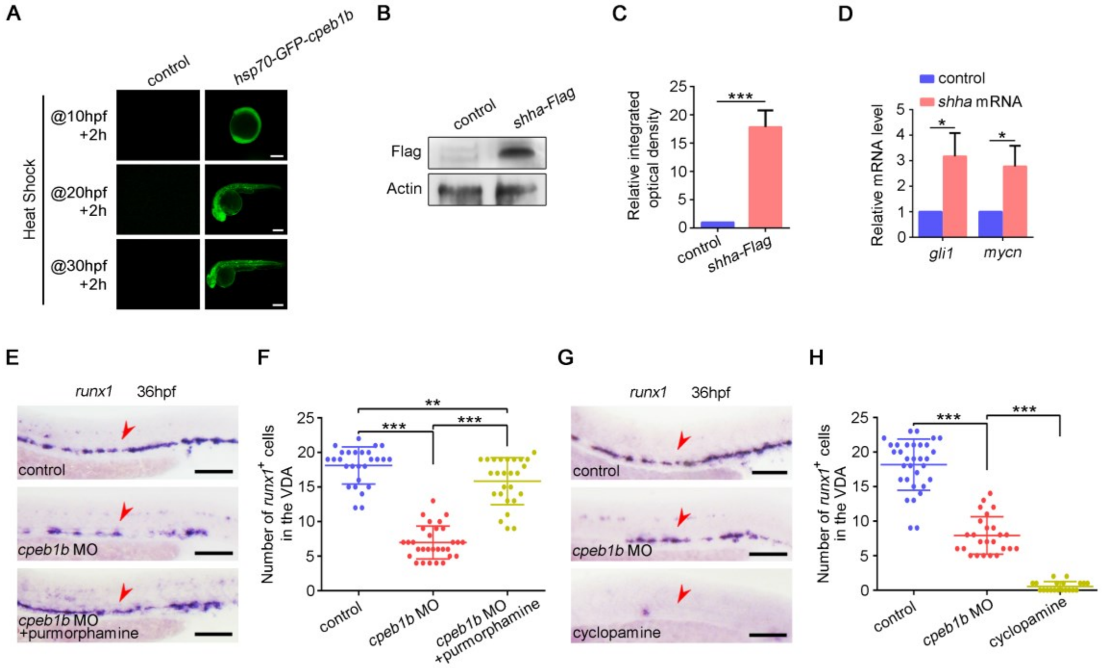

Fig. S3. Cpeb1b regulates Hedgehog signaling. (*A*) Fluorescence microscope imaging shows that GFP expression is detected by 2 hours post heat-shock in *hsp70-GFP-cpeb1b* groups, but not in control groups. Scale bar, 200 µm. (*B*) *Flag*-tagged *shha* mRNA was injected into one-cell stage embryos. The protein level of Shha-Flag and Actin was examined by WB. (*C*) Quantification of relative Shha-Flag protein level using gray analysis. Error bar, mean ± S.D., *P* value was calculated by Student's t-test, $^{***}P < 0.001$. (*D*) Relative mRNA level of Hedgehog signaling downstream gene *gli1* and *mycn* in control and *shha* mRNA overexpression groups examined by qRT-PCR. Error bar, mean ± S.D., *P* value was calculated by Student's t-test, $^{*}P < 0.05$. (*E*) Examination of *runx1* expression in the control, *cpeb1b* morphants, and purmorphamine (25 µM) treated *cpeb1b* morphants by WISH. (*F*) Statistical analysis of the WISH. Error bar, mean ± S.D., *P* value was calculated by Student's t-test, $^{**}P < 0.01$, $^{***}P < 0.001$. (*G*) Examination of *runx1* expression in the control, *cpeb1b* morphants, and cyclopamine (100 µM) treated embryos by WISH. (*H*) Statistical analysis of the WISH. Error bar, mean ± S.D., *P* value was calculated by Student's t-test, $^{***}P < 0.001$.

Figure S4

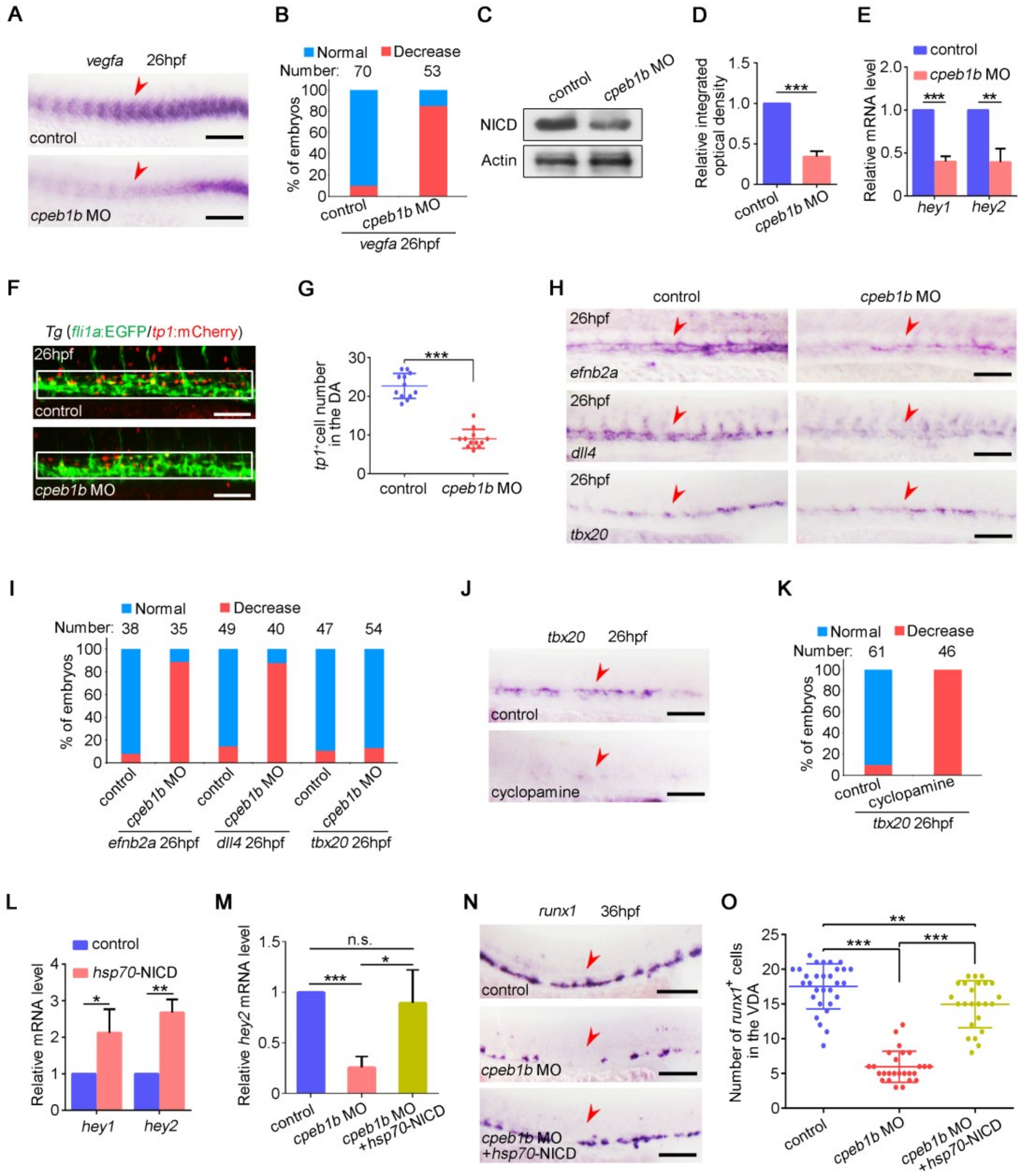

Fig. S4. Cpeb1b regulates Hedgehog-Vegf-Notch pathway. (*A*) Examination of *vegfa* expression in the control and *cpeb1b* morphants at 26 hpf by WISH. (*B*) Quantification of the WISH. (*C*) Protein level of NICD and Actin in the control and *cpeb1b* morphants examined by WB. (*D*) Quantification of relative NICD protein level using gray analysis. Error bar, mean ± S.D., *P* value was calculated by Student's t-test, ****P* < 0.001. (*E*) Relative mRNA level of Notch downstream gene *hey1* and *hey2* in the control and *cpeb1b* morphants examined by qRT-PCR. Error bar, mean ± S.D., *P* value was calculated by Student's t-test, ***P* < 0.01, ****P* < 0.001. (*F*) Confocal imaging shows the *fli1a*$^{+}$*tp1*$^{+}$ Notch-active cells in the VDA region of the control and

cpeb1b morphants at 26 hpf. Scale bar, 100 µm. (*G*) Quantification of *fli1a*$^+$*tp1*$^+$ Notch-active cells in the VDA region. Error bar, mean ± S.D., *P* value was calculated by Student's t-test, ***P < 0.001. (*H*) Expression of *efnb2a*, *dll4*, and *tbx20* in the control and *cpeb1b* morphants at 26 hpf examined by WISH. The red arrowheads denote target cells. Scale bar, 100 µm. (*I*) Quantification of the WISH. (*J*) Examination of *tbx20* expression in control and cyclopamine (100 µM) treated embryos by WISH. (*K*) Quantification of the WISH. (*L*) Relative mRNA level of *hey1* and *hey2* in control and NICD overexpression groups at 26 hpf examined by qRT-PCR. NICD overexpression was carried out by *hsp70*-NICD heat-shock at 14 hpf. Error bar, mean ± S.D., *P* value was calculated by Student's t-test, *$P < 0.05$, **$P < 0.01$. (*M*) Relative mRNA level of *hey2* in the control, *cpeb1b* morphants, and NICD-overexpressed *cpeb1b* morphants at 26 hpf examined by qRT-PCR. Error bar, mean ± S.D., *P* value was calculated by Student's t-test, n.s.: no significance, *$P < 0.05$, ***$P < 0.001$. (*N*) Examination of *runx1* expression in the control, *cpeb1b* morphants, and NICD-overexpressed *cpeb1b* morphants by WISH. (*O*) Statistical analysis of the WISH. Error bar, mean ± S.D., *P* value was calculated by Student's t-test, **$P < 0.01$, ***$P < 0.001$.

Figure S5

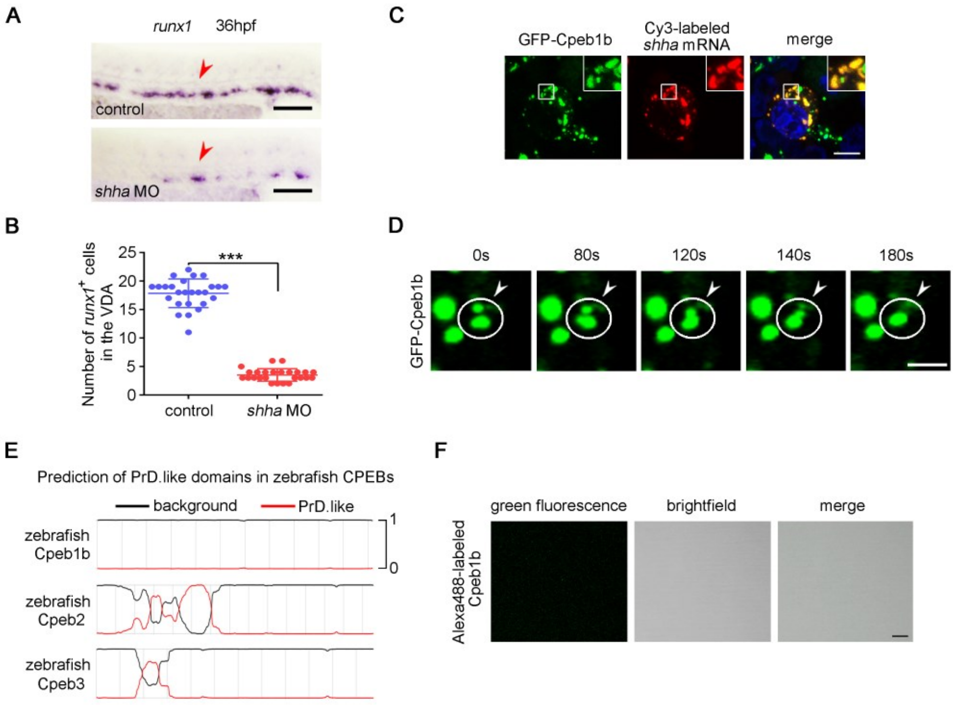

Fig. S5. Cpeb1b itself is not able to perform phase separation. (*A*) Examination of the expression of *runx1* in the control and *shha* morphants at 36 hpf by WISH. The red arrowheads denote HSPCs. Scale bar, 100 μm. (*B*) Statistical analysis of the WISH. Error bar, mean ± S.D., ****P*<0.001. (*C*) Confocal imaging shows colocalization of GFP-Cpeb1b and Cy3-labeled *shha* mRNA in the cytoplasm of HEK293T cells. Scale bar, 10 μm. (*D*) Time-lapse imaging shows the fusion of GFP-Cpeb1b droplets in HEK293T cells. In these representative snapshots, four condensates were showed, and two condensates fused. The lapsed time was 180 s. Scale bar, 2 μm. (*E*) The prion-like domains were predicted by the Prion-Like Amino Acid Composition (PLAAC) algorithm. (*F*) In vitro condensates formation assay for Alexa488-labeled Cpeb1b. The 5 μM Cpeb1b protein in buffer containing 150 mM KCl. Scale bar, 10 μm.

Figure S6

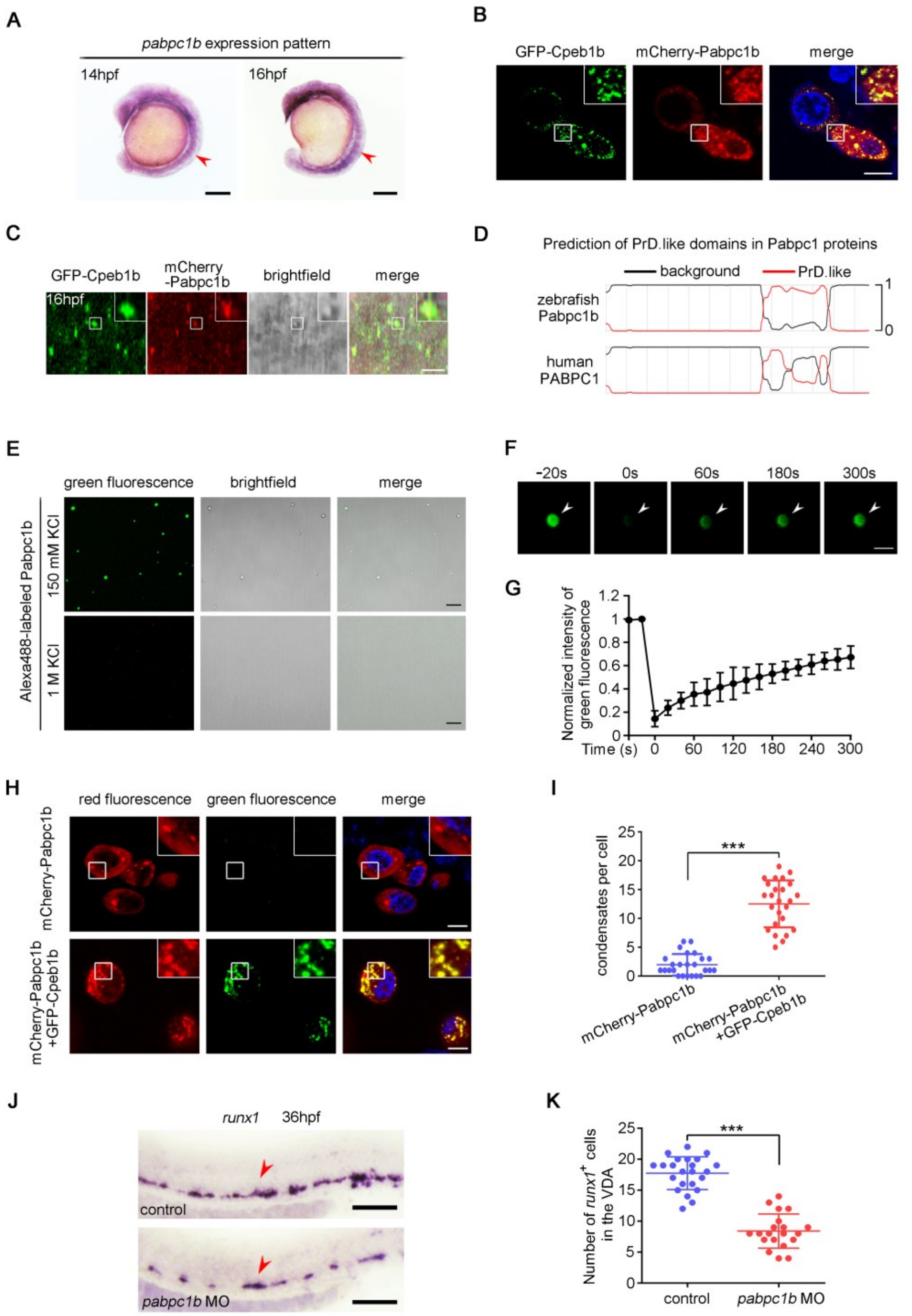

Fig. S6. Cpeb1b promotes Pabpc1b phase separation. (*A*) Examination of *pabpc1b* expression pattern by WISH. (*B*) Confocal imaging shows co-localization of GFP-Cpeb1b and mCherry-Pabpc1b in the cytoplasm of HEK293T cells. Scale bar, 10 μm. (*C*) Confocal imaging shows co-localization of GFP-Cpeb1b and mCherry-Pabpc1b in the notochord of 16 hpf embryos. Scale bar, 10 μm. (*D*) The prion-like domains were predicted by the PLAAC algorithm. (*E*) In vitro condensates formation assay for Alexa488-labeled Pabpc1b. Scale bar, 10 μm. (*F*) The

representative images of fluorescence recovery of Pabpc1b droplets. Scale bar, 2 μm. (*G*) Relative quantification of fluorescence recovery kinetics of Pabpc1b droplets. Three condensates were tested, and three condensates recovered. The lapsed time was 320 s. The black curve shows the mean ± S.D. (n=3). (*H*) Confocal imaging shows Pabpc1b droplets in HEK293T cells. Scale bar, 10 μm. (*I*) Quantification of the number of droplets per cell in fluorescence images. Error bar, mean ± S.D.. *P* value was calculated by Student's t-test, ***P < 0.001. (*J*) Examination of the expression of *runx1* in the control and *pabpc1b* morphants by WISH. The red arrowheads denote HSPCs. Scale bar, 100 μm. (*K*) Statistical analysis of the WISH. Error bar, mean ± S.D., ***P<0.001.

Figure S7

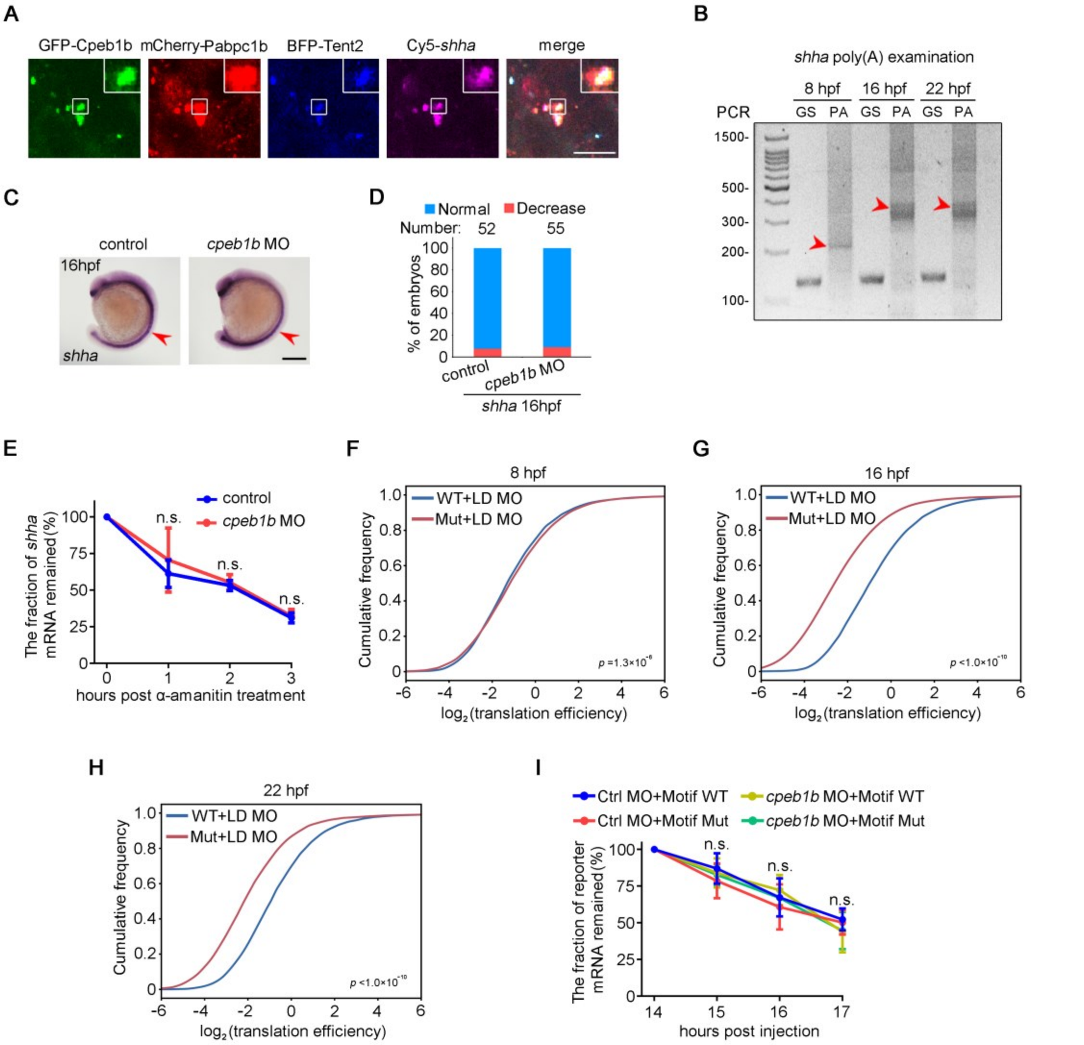

Fig. S7. Cpeb1b-mediated cytoplasmic polyadenylation of *shha* is important for HSPC production. (*A*) The confocal imaging showed the co-localization of GFP-Cpeb1b, mCherry-Pabpc1b, BFP-Tent2, and Cy5-*shha* mRNA in the cytoplasmic condensates of HEK293T cells. (*B*) Examination of the poly(A) length of *shha* mRNA at different developmental stages by PAT assay. The PCR products were detected by gel electrophoresis. The red arrowheads denote smear bands from poly(A) PCR products. GS: gene-specific products, PA: poly(A) tail-included products. (*C*) Examination of the *shha* mRNA expression in the control and *cpeb1b* morphants by WISH. (*D*) Quantification of the WISH. (*E*) The control and *cpeb1b* morphants were treated with α-amanitin mainly for inhibition of polymerase II-mediated transcription, and the relative *shha* mRNA level was determined by qRT-PCR at the indicated time intervals. For α-amanitin treatment, 0.4 ng α-amanitin was injected into the yolk region of 14 hpf embryos. The *shha* mRNA level was

normalized to 18s rRNA, and the relative RNA level at 0 hour post α-amanitin treatment was set as 100%. (*F*) Cumulative distribution of the translation efficiency in the low-dose *cpeb1b* MO injected WT and *cpeb1b* mutant embryos at 8 hpf examined by ribosome profiling. *P* value was calculated by the Kruskal Wallis test. (*G*) Cumulative distribution of the translation efficiency in the low-dose *cpeb1b* MO injected WT and *cpeb1b* mutant embryos at 16 hpf examined by ribosome profiling. (*H*) Cumulative distribution of the translation efficiency in the low-dose *cpeb1b* MO injected WT and *cpeb1b* mutant embryos at 22 hpf examined by ribosome profiling. (*I*) The relative reporter mRNA levels were determined by qRT-PCR at the indicated time intervals in the control and *cpeb1b* morphants by using primers that target *shha* CDS and *Flag* sequence in the reporter. The reporter mRNA level was normalized to 18s rRNA, and the relative RNA level at 14-hour post injection was set as 100%.

Figure S8

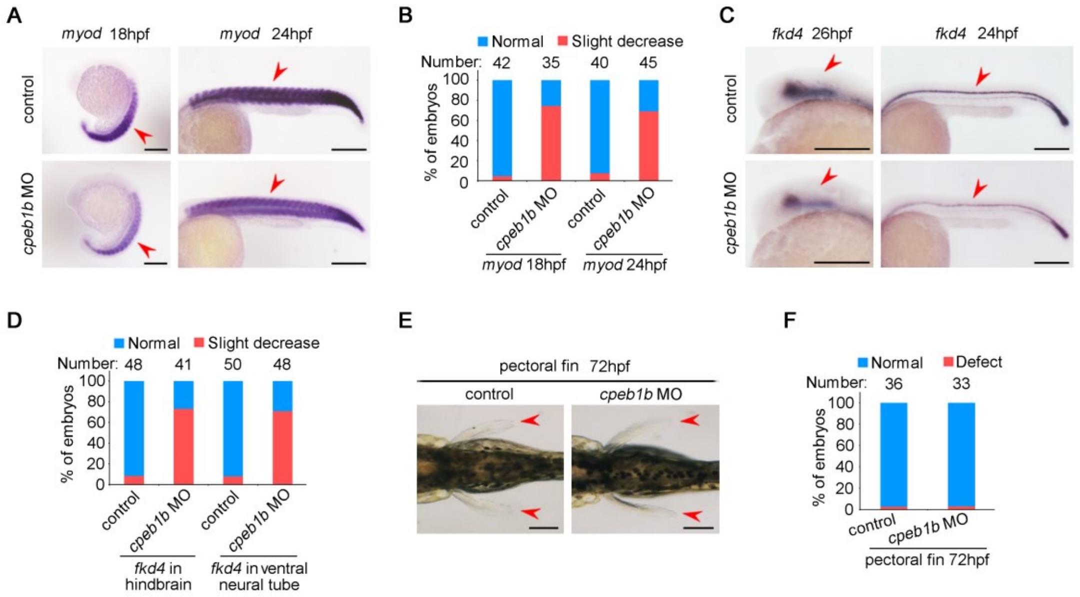

Fig. S8. Cpeb1b-deficiency mildly affects the nervous system and somite, but not pectoral fin. (*A*) Examination of *myod* expression at 18- and 24 hpf in the control and *cpeb1b* morphants by WISH. Scale bar, 100 µm. (*B*) Quantification of the WISH. (*C*) Examination of *fkd4* expression at 24- and 26 hpf in the control and *cpeb1b* morphants by WISH. Scale bar, 100 µm. (*D*) Quantification of the WISH. (*E*) Examination of pectoral fin at 72 hpf in the control and *cpeb1b* morphants. Scale bar, 100 µm. (*F*) Quantification of pectoral fin phenotypes.

Supplemental References

1. Bertrand JY*, et al.* (2010) Haematopoietic stem cells derive directly from aortic endothelium during development. *Nature* 464(7285):108-111.
2. Lawson ND & Weinstein BM (2002) In vivo imaging of embryonic vascular development using transgenic zebrafish. *Dev Biol* 248(2):307-318.
3. North TE*, et al.* (2007) Prostaglandin E2 regulates vertebrate haematopoietic stem cell homeostasis. *Nature* 447(7147):1007-1011.
4. Parsons MJ*, et al.* (2009) Notch-responsive cells initiate the secondary transition in larval zebrafish pancreas. *Mech Dev* 126(10):898-912.
5. Wei W*, et al.* (2008) Gfi1.1 regulates hematopoietic lineage differentiation during zebrafish embryogenesis. *Cell Res* 18(6):677-685.
6. Yang Y*, et al.* (2021) A single-cell-resolution fate map of endoderm reveals demarcation of pancreatic progenitors by cell cycle. *Proc Natl Acad Sci U S A* 118(25).
7. Kwan KM*, et al.* (2007) The Tol2kit: a multisite gateway-based construction kit for Tol2 transposon transgenesis constructs. *Dev Dyn* 236(11):3088-3099.
8. Murphey RD, Stern HM, Straub CT, & Zon LI (2006) A chemical genetic screen for cell cycle inhibitors in zebrafish embryos. *Chem Biol Drug Des* 68(4):213-219.
9. Hondele M*, et al.* (2019) DEAD-box ATPases are global regulators of phase-separated organelles. *Nature* 573(7772):144-148.
10. Lin Y, Protter DS, Rosen MK, & Parker R (2015) Formation and Maturation of Phase-Separated Liquid Droplets by RNA-Binding Proteins. *Mol Cell* 60(2):208-219.
11. Renaud O, Herbomel P, & Kissa K (2011) Studying cell behavior in whole zebrafish embryos by confocal live imaging: application to hematopoietic stem cells. *Nat Protoc* 6(12):1897-1904.
12. Kusov YY, Shatirishvili G, Dzagurov G, & Gauss-Muller V (2001) A new G-tailing method for the determination of the poly(A) tail length applied to hepatitis A virus RNA. *Nucleic Acids Res* 29(12):E57-57.
13. Martin M (2011) Cutadapt removes adapter sequences from high-throughput sequencing reads. *EMBnet. journal* 17(1):10-12.
14. Bolger AM, Lohse M, & Usadel B (2014) Trimmomatic: a flexible trimmer for Illumina sequence data. *Bioinformatics* 30(15):2114-2120.
15. Kim D, Langmead B, & Salzberg SL (2015) HISAT: a fast spliced aligner with low memory requirements. *Nat Methods* 12(4):357-360.
16. Li H*, et al.* (2009) The Sequence Alignment/Map format and SAMtools. *Bioinformatics* 25(16):2078-2079.
17. Love MI, Huber W, & Anders S (2014) Moderated estimation of fold change and dispersion for RNA-seq data with DESeq2. *Genome Biol* 15(12):550.
18. Zhou Y*, et al.* (2019) Metascape provides a biologist-oriented resource for the analysis of systems-level datasets. *Nat Commun* 10(1):1523.
19. Shi B*, et al.* (2020) RNA structural dynamics regulate early embryogenesis through controlling transcriptome fate and function. *Genome Biol* 21(1):120.
20. Trapnell C, Pachter L, & Salzberg SL (2009) TopHat: discovering splice junctions with RNA-Seq. *Bioinformatics* 25(9):1105-1111.
21. Zhang Y*, et al.* (2008) Model-based analysis of ChIP-Seq (MACS). *Genome Biol* 9(9):R137.
22. Quinlan AR & Hall IM (2010) BEDTools: a flexible suite of utilities for comparing genomic

features. *Bioinformatics* 26(6):841-842.
23. Calviello L*, et al.* (2016) Detecting actively translated open reading frames in ribosome profiling data. *Nature methods* 13(2):165-170.
24. Langmead B, Trapnell C, Pop M, & Salzberg SL (2009) Ultrafast and memory-efficient alignment of short DNA sequences to the human genome. *Genome Biol* 10(3):R25.
25. Trapnell C*, et al.* (2010) Transcript assembly and quantification by RNA-Seq reveals unannotated transcripts and isoform switching during cell differentiation. *Nature Biotechnology* 28(5):511-515.
